# Comparative Analysis of the Circular Transcriptome in Muscle, Liver, and Testis in Three Livestock Species

**DOI:** 10.3389/fgene.2021.665153

**Published:** 2021-05-10

**Authors:** Annie Robic, Chloé Cerutti, Christa Kühn, Thomas Faraut

**Affiliations:** ^1^INRAE, ENVT, GenPhySE, Université de Toulouse, Castanet-Tolosan, France; ^2^Institute Genome Biology, Leibniz Institute for Farm Animal Biology (FBN), Dummerstorf, Germany; ^3^Faculty of Agricultural and Environmental Sciences, University of Rostock, Rostock, Germany

**Keywords:** circular RNA, annotation, sub-exonic circRNA, intronic circRNAs, parent genes, circular transcriptome, exonic circRNA

## Abstract

Circular RNAs have been observed in a large number of species and tissues and are now recognized as a clear component of the transcriptome. Our study takes advantage of functional datasets produced within the FAANG consortium to investigate the pervasiveness of circular RNA transcription in farm animals. We describe here the circular transcriptional landscape in pig, sheep and bovine testicular, muscular and liver tissues using total 66 RNA-seq datasets. After an exhaustive detection of circular RNAs, we propose an annotation of exonic, intronic and sub-exonic circRNAs and comparative analyses of circRNA content to evaluate the variability between individuals, tissues and species. Despite technical bias due to the various origins of the datasets, we were able to characterize some features (i) (ruminant) liver contains more exonic circRNAs than muscle (ii) in testis, the number of exonic circRNAs seems associated with the sexual maturity of the animal. (iii) a particular class of circRNAs, sub-exonic circRNAs, are produced by a large variety of multi-exonic genes (protein-coding genes, long non-coding RNAs and pseudogenes) and mono-exonic genes (protein-coding genes from mitochondrial genome and small non-coding genes). Moreover, for multi-exonic genes there seems to be a relationship between the sub-exonic circRNAs transcription level and the linear transcription level. Finally, sub-exonic circRNAs produced by mono-exonic genes (mitochondrial protein-coding genes, ribozyme, and sno) exhibit a particular behavior. Caution has to be taken regarding the interpretation of the unannotated circRNA proportion in a given tissue/species: clusters of circRNAs without annotation were characterized in genomic regions with annotation and/or assembly problems of the respective animal genomes. This study highlights the importance of improving genome annotation to better consider candidate circRNAs and to better understand the circular transcriptome. Furthermore, it emphasizes the need for considering the relative “weight” of circRNAs/parent genes for comparative analyses of several circular transcriptomes. Although there are points of agreement in the circular transcriptome of the same tissue in two species, it will be not possible to do without the characterization of it in both species.

## Introduction

The identification and functional characterization of all transcripts in livestock species is one of the major goals of the consortium for the Functional Annotation of Animal Genomes (FAANG^1^). An animal genome contains 20,000 to 30,000 genes but only a subset of these genes produce transcripts in a given tissue. Developments in high-throughput RNA-seq technology have enabled a deeper understanding of gene expression functions. The classical approach for studying the transcriptome uses the mRNA-seq protocol (sequencing of polyadenylated RNAs). A large number of mRNA-seq studies have demonstrated for example that part of the associated genes are transcribed in a tissue specific manner (Soumillon et al., 2013). However, datasets generated by mRNA-seq contain only a part of the transcripts. To overcome this drawback, it is possible to sequence RNAs after depletion of ribosomal sequences (total-RNA-seq) (Chen et al., 2020). Studies using the total-RNA-seq protocol have shown that a large number of protein-coding genes, long non-coding (lnc) RNA genes and intergenic elements are expressed in a tissue-specific manner (Soumillon et al., 2013; Clark et al., 2017; Yang et al., 2020).

Since 2012, advances in high throughput sequencing revealed the presence of circular RNAs (circRNAs) in total-RNA-seq datasets in addition to linear transcripts (Salzman et al., 2012). CircRNAs are probably a natural by-product of the transcription process in all eukaryotes (see Kristensen et al. (2019) for a review). To study circRNAs, it is important to identify the gene that is likely to generate the considered circRNA alongside the linear transcripts already described, namely the parent gene. The majority of studies have focused on exonic circRNA that are generated by the circularization of one or several exons through a back splicing process: the end of an exon is joined to the beginning of an upstream exon (Kristensen et al., 2019; Li et al., 2019). Exonic circRNAs can be produced by coding or non-coding genes (Salzman et al., 2012; Barrett et al., 2017; Robic et al., 2020) and by some snRNA genes (Kaur et al., 2018; Robic et al., 2020). Two types of circRNAs can be derived from intronic sequences (see Robic and Kühn (2020) for a review): (1) when intron lariats escape degradation due to the failure of intron debranching (Zhang et al., 2013), they may become circRNA precursors of lariat-derived circRNAs and (2) for very rare introns we can observe the circularization of the entire intronic sequences as intron circle (Taggart et al., 2017). These two types of intron derived circRNAs can be grouped as “intronic circRNAs.” Sub-exonic circRNAs have been characterized as including only a part of the exon of some mono-exonic genes (Robic et al., 2020). Up to now, only intronic circRNA from protein-coding genes and only sub-exonic circRNAs from small-non-coding (snc) RNA have been reported in pigs (Robic et al., 2020). Understanding to what extent, these different subclasses of circRNAs are produced and what kind of genes are able to produce them is a question of interest.

In 2013, circRNAs were shown to have functional relevance (Jeck et al., 2013; Memczak et al., 2013) as reviewed by (Chen, 2020; Xiao et al., 2020). Since the landmark discovery of ciRS-7/CDR1as functioning as a miR-7 sponge in Hansen et al. (2013), a lot of studies focused on circRNAs action as microRNA decoys. However, as circRNA research expands, many divergent views have emerged (reviewed by Li et al., 2019) and our understanding of circRNAs, their production and their function, remains limited. The diversity of functions assigned to circRNAs is very large but concerns only some circRNAs. For example, recent studies have highlighted that the presence of a particular circRNA from *SLC45A4* is essential to keep neural cells in a progenitor state in the mammalian brain (Suenkel et al., 2020). Recently, the regulatory functions of two circRNAs produced respectively from a mitochondrial gene (Zhao et al., 2020) and from an intron (Das et al., 2020; Stoll et al., 2020) were characterized. These studies have also underlined the need to work on the conservation of circRNAs, and beyond exonic circRNAs.

The identification of circular RNAs in highly divergent species raises interesting questions about their evolutionary history, and functions (Wang et al., 2014; Ji et al., 2019; Zucko and Boris-Lawrie, 2020). Li et al. (2019), who reviewed this topic, reported wide discrepancies: some studies claimed that circRNAs are evolutionarily highly conserved molecules, while others believe they are species-specific. For our study, we take advantage of functional datasets produced within partners and also the FAANG consortium to study circular RNA in farm animals (cattle, pig, sheep). We studied the pervasiveness of circRNA transcription in three tissues (skeletal muscle, liver, and testis). As the transcriptomes of these three tissues present specific features (Yang et al., 2020), this choice seemed to us pertinent to compare and analyze circRNA production.

## Materials and Methods

### Data Collection

For this study, we collected total-RNA-seq data produced by our groups and others from the literature. The whole dataset contains 33 bovine tissues, 15 ovine tissues and 19 pig tissues (Table 1). The considered datasets are originating from three SRA projects for bovine tissue and from four SRA projects for porcine tissues. All the ovine datasets were generated at Roslin Institute in a unique SRA biological project PRJEB19199 (Clark et al., 2017). We achieved to consider at least 70 giga bases (gb) of sequencing data for each tissue in a given species. In the following, a batch is defined as a collection of datasets from a single tissue of animals from the same species, same sex, same age, and originating from a unique SRA project. In Table 1, these datasets are clustered in 17 batches and one singleton. The dataset ssc_testis_1 was excluded from the batch, which constituted datasets from the SRA project PRJNA506525, because of its atypical behavior regarding the production of circRNAs (Robic et al., 2019). Among the 48 animals from the SRA project PRJEB34570 (Nolte et al., 2019), we chose six males and six females to obtain two batches balanced on known physiological traits. For bovine testis (Gao et al., 2019), we selected datasets from bulls at 13 months of age (bta_testis_4-6), which is assumed an age at the end of puberty (Rawlings et al., 2008; McGowan et al., 2018), to represent data from pubertal testis.

**TABLE 1:** Samples characteristics.

Datasets	Species	Tissue	Age animal	Sex animal	Breed		Reads PE (bp)	SRA project	gb
bta_liver_1-6 ^(1)^	cattle	liver	18 months	male	Charolais X Holstein-F2	FBN (Nolte et al., 2019)	2 × 100	PRJEB34570	68.1
bta_liver_7-12 ^(1)^	cattle	liver	3.5 years	female	Charolais X Holstein-F2	FBN (Nolte et al., 2019)	2 × 100	PRJEB34570	68.3
bta_liver_13-15	cattle	liver	”adult”	male		EMBL-2017	2 × 10	PRJEB13074	26.4
bta_muscle_1-6 ^(1)^	cattle	muscle	18 months	male	Charolais X Holstein-F2	FBN (Nolte et al., 2019)	2 × 100	PRJEB34570	55.1
bta_muscle_7-12 ^(1)^	cattle	muscle	3.5 years	female	Charolais X Holstein-F2	FBN (Nolte et al., 2019)	2 × 100	PRJEB34570	56.5
bta_testis_1-3	cattle	testis	2 days	male	Angus	Yangling (Gao et al., 2019)	2 × 150	PRJNA47564	46.5
bta_testis_4-6	cattle	testis	13 months	male	Angus	Yangling (Gao et al., 2019)	2 × 150	PRJNA47564	43.9
ssc_liver_5-7 ^(2)^	pig	liver	2 years	male		EMBL-2019	2 × 150	PRJEB33381	46.0
ssc_liver_8-10	pig	liver	”adult”	male		EMBL-2017	2 × 100	PRJEB13074	26.6
ssc_muscle_2-4 ^(2)^	pig	muscle	2 years	male		EMBL-2019	2 × 150	PRJEB33381	87.8
ssc_testis_1 ^(3) (4)^	pig	testis	6 months	male	Pietrain (Pi)	INRAE (Robic et al., 2019)	2 × 125	PRJNA506525	17.3
ssc_testis_2-7 ^(4)^	pig	testis	6 months	male	3 Pi & 3Pi X Large White	INRAE (Robic et al., 2019)	2 × 125	PRJNA506525	115.2
ssc_testis_8-10 ^(2)^	pig	testis	2 years	male		EMBL-2019	2 × 150	PRJEB33381	56.6
oar_liver_1-3 ^(1)^	sheep	liver	2 years	male	Scot. Blackface x Texel	Roslin (Clark et al., 2017)	2 × 125	PRJEB19199	90.8
oar_liver_4-6 ^(1)^	sheep	liver	2 years	female	Scot. Blackface x Texel	Roslin (Clark et al., 2017)	2 × 125	PRJEB19199	90.8
oar_muscle_1-3 ^(1)^	sheep	muscle	2 years	male	Scot. Blackface x Texel	Roslin (Clark et al., 2017)	2 × 125	PRJEB19199	99.4
oar_muscle_4-6 ^(1)^	sheep	muscle	2 years	female	Scot. Blackface x Texel	Roslin (Clark et al., 2017)	2 × 125	PRJEB19199	91.8
oar_testis_1-3 ^(1)^	sheep	testis	2 years	male	Scot. Blackface x Texel	Roslin (Clark et al., 2017)	2 × 125	PRJEB19199	92.9

*Seven teen groups of datasets were collected, combining total RNAseq generated by our groups and others from the literature. Only groups of datasets generated by sequencing stranded RNA from healthy animals and containing at least three datasets produced in parallel were selected. ^1^The animals from [bta_liver_1-6 and bta_muscle_1-6], [bta_liver_7-12 and bta_muscle_7-12], [oar_liver_1-6 and oar_muscle_1-6], and [oar_liver_4-6, oar_muscle_4-6 and oar_testis_1-3] were the same and were presented in the same order. ^2^The three animals from [ssc_liver_5-7, ssc_muscle_2-4 and ssc_testis_8-10] were the same but they were not presented in the same order. ^3^We chose to not associate the dataset ssc_testis_1 to other datasets from the SRA project PRJNA506525 because we know that this datasets is an outlier dataset in its origin group. ^4^Datasets ssc_testis_1-7 were obtained from corresponding animals previously called Animal−31, −05, −54, −16, −65, −73, and −93 in Robic et al. (2019).*

To analyze the impact of read length on the circular RNA detection, we produced 6 new datasets with 2 × 100 bp PE (Paired- End) sequencing from the 6 datasets from cattle testis samples, which had been previously sequenced for 2 × 150 bp PE.

### Reads Mapping

The RNA-seq reads were aligned to the following genome reference assemblies: ARS-UCD1.2 (GCA_002263795.2), Oar_rambouillet_v1.0 (GCA_002742125.1), Sscrofa11.1 (GCA_000003025.6) for cow, sheep and pig respectively. We used the gene annotation (v-101) provided by Ensembl (Ensembl-Websites: Cattle, 2021; Pig, 2021; Sheep, 2021).

RNA-seq reads were mapped to the genome reference assemblies using the rapid splice-aware read mapper Spliced Transcripts Alignment (STAR) (Dobin et al., 2013). Two alignment modes were considered, single-end alignments (STAR-SE option, mates of each pair were mapped independently) and paired-end alignments (STAR-PE option). STAR was used with the previously proposed parameters (Cheng et al., 2016) that enable the highlighting of chimeric reads with only two segments and with a minimal size for the smallest mapped segment of 15 bp.

### CircRNA Detection and Annotation

The first step in the detection of circRNAs is the identification of reads containing a circular junction (see Gao and Zhao, 2018 for a review). The analysis of these reads allows to describe each circRNA by the two points involved in the circular junction (the genomic boundaries of the circularized transcript: two genomic coordinates) and the strand. The second step of the characterization of circRNAs is their annotation.

#### Standard CircRNA Detection and Quantification

The first approach for detecting circRNAs used the combination of circRNAs detected by CIRCexplorer2 (CE2, Zhang et al., 2016) and CIRI2 (Gao et al., 2015, 2018) which have become reference tools for the identification of exonic circRNAs (Zeng et al., 2017; Gao and Zhao, 2018; Hansen, 2018; Dodbele et al., 2021). CE2 is able to use several aligners and our choice was to use CE2 associated to STAR-PE (Dobin et al., 2013) alignment mode (Zhang et al., 2016). It is important to note that we have chosen more stringent parameters for the alignment performed with STAR-PE than those suggested by Zhang et al. (2016) (see above) for the detection of chimeric reads. As CE2 identifies reads containing a circular junction within those reads that STAR calls “chimeric reads” (CR), we will call these reads “circular chimeric reads” (hereafter CCRs). CIRI2 (Gao et al., 2018) is based on the bwa-mem aligner (Au et al., 2016) together with a dedicated approach to align unmapped segments. CIRI2 was used to identify the reads containing circular junctions with default parameters. Reads containing a circular junction are called BSJ (“back-spliced junction”) reads by CIRI2.

All circular RNAs identified by CE2 as generated from backsplicing of two described exons were considered as exonic circRNAs. Those annotated as “ciRNA” correspond to circRNAs localized entirely in intronic sequences and with the circRNA 5′ junction site corresponding to the intron donor site. Although the term “ciRNA” is the one proposed for intronic circular RNA by Zhang et al. (2013), the location of the 3′ junction of these circRNAs must be analyzed before to consider them as intronic circRNA (Robic and Kühn, 2020).

Only circRNAs detected by both CE2 and CIRI2 were considered for quantification as suggested previously by Hansen (2018). A CIRI2 formatted list of circRNAs was provided to CIRIquant (Zhang et al., 2020) to obtain an accurate quantification of circRNAs. The quantitative measure is the number of BSJ reads provided by CIRIquant. The expression measure for each parent gene is simply the sum of expression measure of the circRNAs it produces. In order to obtain, for each circRNA of each parent gene, an average expression for the tissue, the average expression over all animals was computed after normalization by animal (TMM normalization provided by edgeR, Robinson et al., 2010).

#### Detection of Orthologous circRNAs

Orthologous circular RNAs were identified based on nucleotide sequence alignments. Each circRNA is represented by the nucleotide sequence crossing the circular junction point (200bp, 100 bp on each side). Given the close evolutionary between bovine and ovine we have limited the detection of orthologous circRNAs to those two species. All circRNA bovine sequences were aligned to ovine circRNA sequences and reciprocal best hits were considered as orthologous circRNAs. For the parent genes, the orthologs were defined as the Ensembl one-to-one orthologs.

#### Computational Approach for Exhaustive Detection and Characterization of circRNAs Complementary to Standard Tools

In this manuscript, we use CD as an abbreviation for our dedicated approach to detect circRNAs. Our approach to identify reads containing a circular junction is based on split alignment as defined by Gao et al. (2018) and was originally proposed by Memczak et al. (2013). This approach has previously been described (Robic et al., 2020), and we underline only some essential features. The focus of this alternative framework method is limited to selecting reads that are mapped by STAR-SE as CR with only two segments, and where both segments are mapped to the same strand in inverted order. To select CCRs, we extracted information from the tabular file (chimeric.out.junction) provided by STAR, which contains the mapping coordinates of each segment and mapping data (CIGAR). An output file (BED format) containing a list of circRNAs is obtained by clustering of CCR on genomic coordinates. The second part of our approach consists in proposing an annotation for the circRNAs detected. The annotation was performed using the species corresponding gene annotation from Ensembl and in particular, the list of exons and the corresponding list of introns.

For the annotation purpose, we define the following classes: (i) exonic, when both junctions correspond exactly to exon boundaries, where both exons belong to the same gene. (ii) sub-exonic, when both junctions fall strictly within a single exon. (iii) intronic, when both junctions fall within a single intron, the 5′ junction corresponding to the intron donor site and the 3′ junction located not further than 60 bp away from the intron acceptor site (Robic and Kühn, 2020). CircRNA from ribosomal RNA genes were excluded from the list of sub-exonic circRNAs. All circRNAs with a too small genomic size (when the genomic size < (1/2 length of read + 5 nt)) were excluded from the annotation process.

## Results

### Circular RNAs: Detection and Annotation

#### Initial circRNAs Landscape Established by CIRI2 and CIRCexplorer2

The detection pipelines detected on average 8,300 and 16,300 circRNAs per sample for CE2 and CIRI2 respectively. A first analysis on six datasets showed that a very large part of exonic circRNA (>90%) detected by CE2 was also detected by CIRI2, while the fraction of ciRNAs (putative intronic circRNAs, see Materials and Methods) proposed by CE2 and detected also by CIRI2 was less than 2%. These observations underline the fact that the detection of non-exonic circRNAs remains a difficult task and at least subject of debate. As it is common practice (Gong et al., 2020), initially recommended by Hansen (2018), we considered only the circRNAs detected by both tools (Dodbele et al., 2021). Moreover, we retained only circRNAs characterized by at least four reads containing the circular junction, and this threshold was applied after the intersection of CE2 and CIRI2 data (BSJs > = 4). On average and in each of the 66 datasets, 1,957 circRNAs were characterized by CE2 + CIRI2.

**FIGURE 1:**
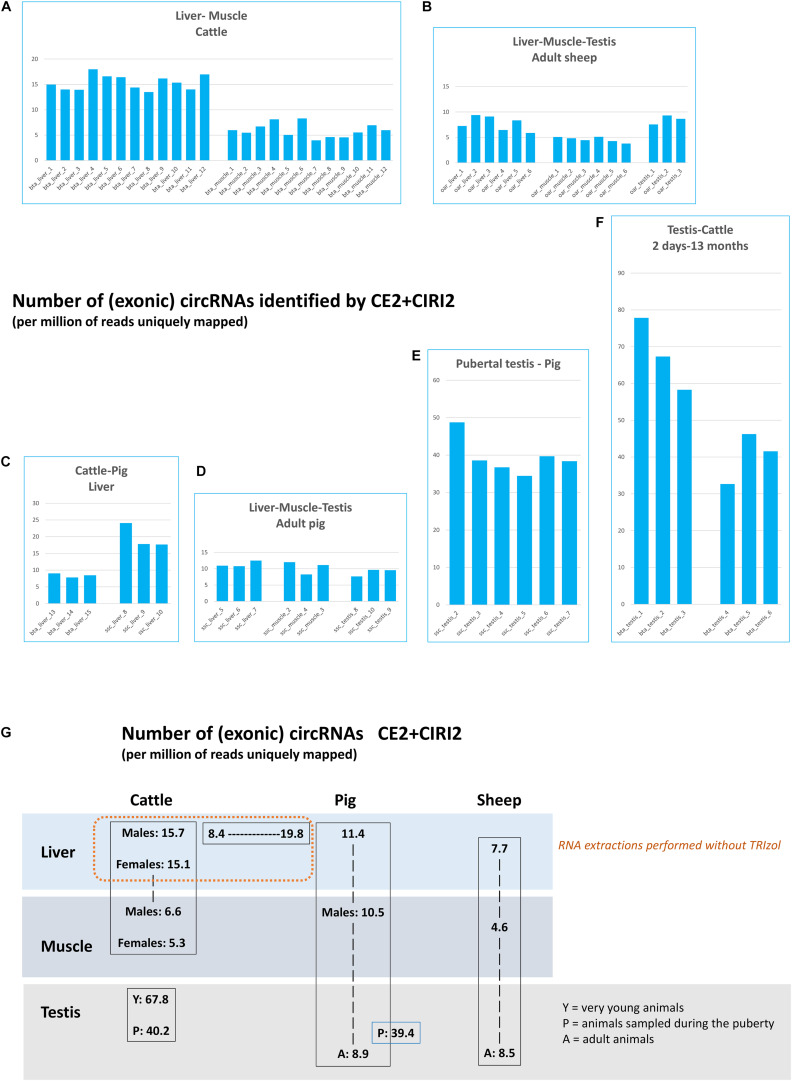
Number of circRNA characterized by CE2 + CIRI2. **(A–G)** These histograms represent the number of circRNA (per million of reads uniquely mapped) characterized jointly by CE2 and CIRI2 and which are detected by at least 4 BSJs (CIRIquant). Histograms are regrouped by SRA projects. **(A)** The two bovine batches produced at FBN. **(B)** the three ovine batches produced at Roslin Institute **(C)** The two batches produced by EMBL in 2017. **(D)** The two batches produced by EMBL in 2019. **(E)** The batch of 6 datasets produced from porcine pubertal testes at INRAE. **(F)** The two batches of bovine testes produced by Yangling University. **(G)** Comparison per tissue and species of the number of exonic circRNAs detected by CE2 + CIRI2 per million of reads uniquely mapped.

Using this strategy, we were able to characterize 12,589 exonic circRNAs and 6 ciRNA in the bovine datasets (Supplementary File 1). For pigs, the statistics were 14,137 and 1, for exonic circRNAs and ciRNAs respectively. For sheep, we found 5,556 exonic circRNAs and 3 ciRNAs. A large variability between datasets in this raw number of circRNAs detected was noted (see Supplementary File 2). Before all further analyses, the number of circRNAs identified in each of the 66 datasets was corrected by the number of uniquely mapped reads by STAR (Supplementary File 2). We compared this normalized number of circRNA in each of the 66 datasets (Figures 1A-F). Since some datasets differ by read length, in order to analyze the impact of read length on the circular detection, we produced six new datasets of 2 × 100 bp PE sequencing from the six bta_testis_1-6 datasets, which had been previously sequenced for 2 × 150 bp PE. The detection of exonic circRNAs was performed by CE2, and we observed a 10 to 20% loss of initial exonic circRNAs with shortened reads (data not shown). This experiment shows that even if the length of the reads has an impact on the detection of exonic circRNAs, this impact is moderate. Therefore, the difference of reads lengths from PE sequencing do not explain the large differences observed between circRNA content of the two batches generated from porcine liver at EMBL in two different SRA projects (ssc_liver_8-10 (Figure 1C) and ssc_liver_5-7 (Figure 1D)). In bovine liver, the number of circRNAs also appeared variable between SRA projects. We observed 13.94 to 17.98 circRNAs per million uniquely mapped reads (per million reads for short) for the 12 first datasets (bta_liver_1-12), and 3.96 to 8.29 for the three others (bta_liver_13-15) (Figures 1A,C), although all samples were sequenced in PE mode with 2 × 100 bp. For bta_liver_1_12, the circRNAs per million reads did not differ much between samples, although the dataset included physiologically very divergent animals, i.e., bulls at the end of fattening and cows at the beginning of lactation. Also in porcine testis, the number of circRNAs seemed very different in datasets produced at EMBL (ssc_testis_8-10) to those produced at INRAE (ssc_testis_2-7). However, in this comparison, the age of the considered animals was different: datasets ssc_testis_8-10 were obtained from adult boars (two years old), while ssc_testis_2-7 originated from pubertal animals (<6 months old).

The number of circRNAs detected in testis of very young bulls seemed higher than in testis from pubertal animals (Figure 1F). As these two datasets were included in the same SRA project and absence of technical bias could be assumed, a statistical analysis was performed (Supplementary File 3), which revealed that the difference in the number of circRNAs was significant (*p*-Value = 0.016). The number of circRNA in testis of pubertal animals appeared similar in bovine and in pigs, and also the number of circRNAs in testis of adult animals displayed a similar level in pigs and sheep (Figure 1G), although these datasets were not generated by the same sequencing lab. This analysis underlines the importance to consider the age (or sexual maturity) of animals for testicular datasets. Since the difference between males and females was not statistically significant for bovine liver and muscle from the same animals (bta_liver_1-6 and _7-12; bta_muscle_1-6 and 7-12), we will no longer differentiate between male and female datasets of this species-tissue combination.

**FIGURE 2:**
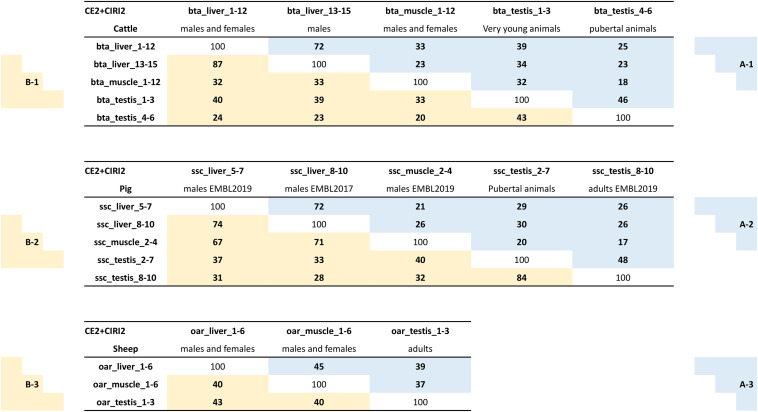
Comparisons circRNAs and parent genes between datasets. The three diagrams depict analyses of exonic circRNAs characterized by CE2 + CIRI2. The number of similarities (same circRNA or same parent gene) found for a comparison between two datasets was reported in a box. **A**-(boxes with a blue background): The expression of a circRNA in a batch has been defined as the sum of the BSJs (normalized counts) observed in the different datasets of this batch. The circRNAs were ranked on their expression to establish the Top-100 of circRNAs expressed in this batch (Lists of Top-100/circRNAs relative to these analyses were reported in Supplementary Table 1). **B**-(boxes with a yellow background): The circRNA expression of genes in a batch has been defined as the sum of the BSJs (normalized counts) from each circRNA produced by this gene observed in the different datasets of this batch. The parent genes were ranked on their circRNA expression to establish the Top-100 of parent genes expressed in this batch (Lists of Top-100/genes relative to these analyses were reported in Supplementary Table 2).

**FIGURE 3:**
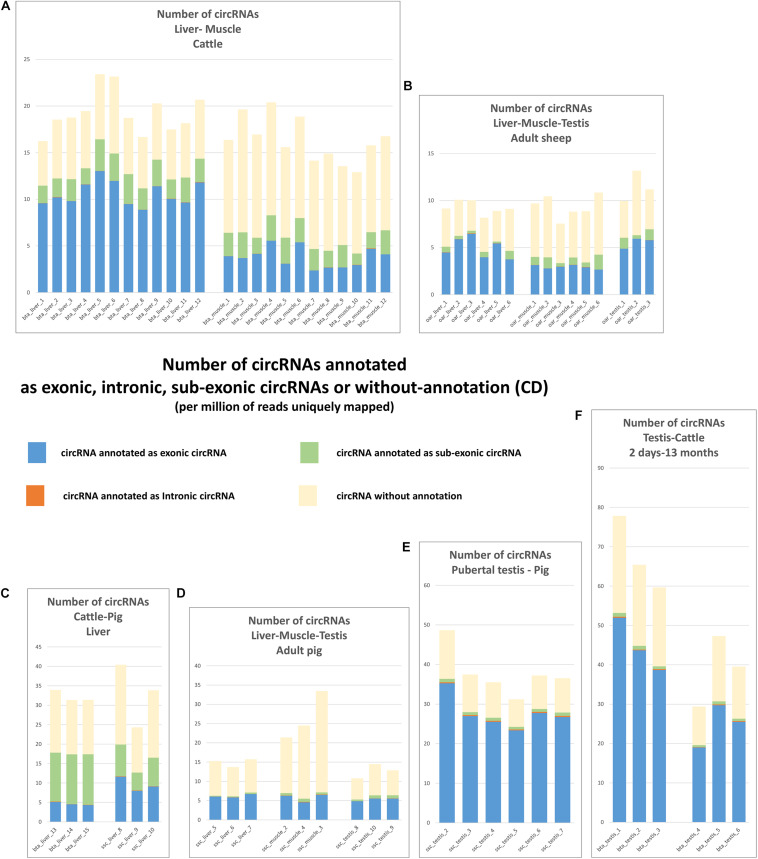
Number of circRNA characterized by CD. These histograms represent the number of circRNA (per million of reads uniquely mapped) which are detected by at least 5 CCRs and annotated as exonic circRNA, intronic circRNAs, sub-exonic or unannotated. Histograms are regrouped by SRA projects. **(A)** The two bovine batches produced at FBN. **(B)** the three ovine batches produced at Roslin Institute **(C)** The two batches produced by EMBL in 2017. **(D)** The two batches produced by EMBL in 2019. **(E)** The batch of 6 datasets produced from porcine pubertal testes at INRAE. **(F)** The two batches of bovine testes produced by Yangling University.

The number of circular junction reads associated with the detection of a circRNA is commonly used to quantify the expression of this circRNA. We chose to perform this quantification by CIRIquant (Zhang et al., 2020), and each circRNA characterized by the CE2 + CIRI2 approach was associated with an expression level measured by the number of BSJs. We considered the sum of the BSJs (corrected by the number of reads uniquely mapped in the dataset) across datasets grouped in the same batch as the expression of this circRNA in the considered batch. We did a ranking of circRNA expressions within each batch; this should enable comparisons of ranking between batches. When we performed a pairwise comparison of the Top-100 most highly expressed circRNAs (Supplementary Table 1) between batches, we found different degrees of overlaps between pairs (Figure 2A in blue). All comparisons were performed within species, even though we also looked at differences/similarities of the statistics between species. Before comparing batches, we compared six pairs of two randomly selected datasets from the batch bta_liver_1-12, and on average 71% of overlaps were observed (six comparisons performed: 63 to 79%). Similar levels of overlap were noted when comparing the two different batches from bovine liver (72%) and between the two batches from porcine liver (72%). These scores showed that the identification of the most highly expressed circRNAs (at least in liver) is not very sensitive to the source of data analyzed for circRNAs characterization. Between muscle and liver, similar levels of overlap were noted in bovine (23-33%) and in pigs (21-26%) (Figures 2A-1,2). In testis, we noted a similar level of overlap between testis from pubertal animals and muscle from other animals (cattle:18% and pigs: 20%). However, the level of overlap between testis and adult muscle seemed to decrease with the age of testis, because we observed a 32% overlap for testis from young animals (cattle), and 17% for testis from adult pigs. These analyses demonstrate the differences in the circRNA expression in testes in relation to the age of the animals. Curiously, the levels of overlap between testis/liver/muscle appeared higher in sheep (Figure 2A-3) than in bovine (Figure 2A-1) or pigs (Figure 2A-2), probably because of differences in genome annotation. These analyses underline once again the importance to consider the age of animals for testicular datasets, but attenuates the importance of the source of datasets with respect to most highly expressed circRNAs. However, it has to be considered that these analyses are restricted almost exclusively to exonic circRNA.

When we examined the circRNAs detected jointly by CE2 and CIRI2 and retained in our analysis (Supplementary List 1), we noted that almost all are exonic circRNAs. As our purpose is to study all types of circRNAs in three tissues of three species, we included a dataset (ssc_testis_1) with a particular circRNA content already explored in previous studies (Robic et al., 2019, 2020). This porcine testicular dataset is known to contain more than 100 intron-derived circRNAs. The major intronic circRNA described in this dataset was a lariat-derived circRNA from the *ATX2NL* gene. CE2 was able to detect circRNAs from the respective *ATX2NL* intron, but with six times less CCRs than previously observed (Robic et al., 2020). CIRI2 did not detect this intronic circRNA, probably because of its small size. CE2 was also able to detect the six intronic circRNAs from the *DNAH17* previously reported (Robic et al., 2019) but again with a lower number of CCRs than expected. These observations confirmed that CE2 is able to detect intronic circRNAs (Das et al., 2020), but as suggested previously (Robic and Kühn, 2020), the strong requests on the two paired-end reads as included in the CE2 algorithm could impair the characterization of intronic circRNAs. The dataset ssc_testis_1 had also been used to describe the first sub-exonic circRNAs (Robic et al., 2020). These sub-exonic circRNAs were characterized by the observation of reads containing a circular junction and spanning parts of the single exon from mono-exonic genes classified as small non-coding RNA. The genes involved were able to produce several, potentially overlapping circRNAs from a single exon. The production of a set of circRNAs by the mono-exonic gene *RMRP* (orthologous gene of porcine/bovine/sheep *RNase_MRP*) was already highlighted in two species (Liu et al., 2020; Robic et al., 2020). This gene was reported as able to produce several dozens of sets of sub-exonic circRNAs in the dataset ssc_testis_1, but only two sub-exonic circRNAs were found in the list of circRNAs provided by CIRI2. From these data, we could conclude that the lists of circRNAs obtained by the conservative approach of CE2 + CIRI2 output fell short with respect to an exhaustive circRNA detection in the three tissues under investigation.

#### Exhaustive Detection and Annotation of circRNAs

As our purpose was to study all types of circRNAs, we used an alternative approach (CD) for the exhaustive detection of circRNA (see section “Materials and Methods”). The next objective was to further annotate the detected circRNAs as either exonic, intronic and sub-exonic, and the remaining as undefined or unannotated. Our objective was not to provide an alternative list of exonic circRNAs to the one established by CE2 + CIRI2, but only to identify a maximum number of exonic circRNA as a prerequisite for an improved, subsequent analysis of the other circRNAs. The criteria to annotate exonic circRNAs were identical to those used by CE2, but for intronic circRNAs we were more stringent (see section “Materials and Methods”). As Liu et al. (2020) suggested that the production of sub-exonic circRNAs was not limited to exons from mono-exonic and non-coding RNA-genes, we integrated in our alternative approach also the detection of sub-exonic circRNAs from all exons: from coding and non-coding genes, from mono-exonic and multi-exonic genes. To avoid including false positives in our analysis, we disregarded very rare circularization events: circRNAs were retained when they were characterized by at least 5 CCRs. This choice was motivated by previous studies using a similar approach for the detection of circRNAs (Robic et al., 2019, 2020). On average and in each of the 66 datasets, CD detected 65,500 circRNAs, and after the application of this threshold, 2,644 circRNAs were retained.

**FIGURE 4:**
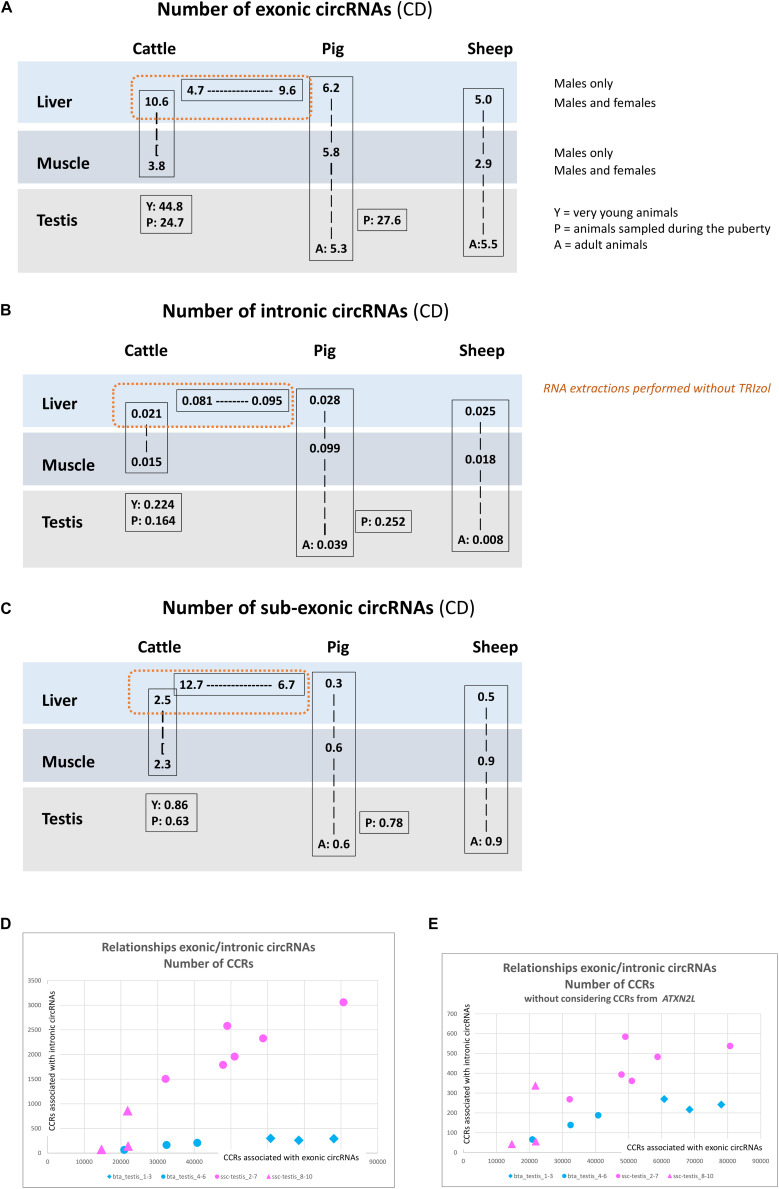
circRNAs detected by CD. **(A)** Number of exonic circRNA detected by CD per million of reads uniquely mapped. **(B)** Number of intronic circRNA detected by CD per million of reads uniquely mapped. **(C)** Number of sub-exonic circRNA detected by CD per million of reads uniquely mapped. **(D,E)** Relationship between the detection of intronic and exonic circRNAs.

Results of the exhaustive detection of circRNA were shown on Figure 3. The number of putative circRNAs detected by CD appeared higher than circRNA detected by CE2 + CIRI2. This difference was particularly marked on the datasets from bovine and porcine muscle (Figures 1A,D, 3A,D). The next step was the characterization of exonic circRNAs and they were indicated in blue on the histograms presented on the Figure 3 (and were listed in Supplementary List 2). For example, 10,351 exonic circRNAs were characterized by CD in 18 porcine datasets (by at least five CCRs in one dataset). Only 50 were never detected by CE2 or CIRI2 (10,358 and 9,940 were detected by CIRI2 and CE2 respectively). The number of exonic circRNAs detected by CD (Figure 4A) appeared consistent with the number of circRNAs (mainly exonic circRNAs) jointly detected by CE2 and CIRI2 (Figure 1G), even though on average, CE2 + CIRI2 detected more circRNA than exonic circRNAs detected by CD.

Next, we proceeded to the identification, in the 67 (66 + 1) datasets, of intronic circular RNA and sub-exonic circRNAs. We detected only a very low number of introns associated with intronic circRNA in several datasets (for example, intronic circRNA were detected for zero to four introns in bovine muscle datasets) (Supplementary List 3), and the dataset ssc_testis_1 turned out again as an outlier (132 introns concerned). In contrast to previous studies, in which only mono-exonic non-coding genes were considered, all sub-exonic circRNA, covered by at least 5 CCRs, for all types of genes (mono- and multi-exonic) were listed. The two ribozyme genes, *RNase_MRP* and *RNaseP_nuc*, are the major small non-coding RNA gene able to produce sub-exonic circRNA. For sub-exonic circRNA assigned to multi-exonic genes, each exon involved produced several, possibly overlapping, sub-exonic circRNAs. We noted that several exons within a particular gene could contribute to the production of sub-exonic circRNAs. For example, we observed sub-exonic circRNAs from the nine exons of *FGB* and from 11 out of the 15 exons of the *ALB* gene in bovine liver (Supplementary List 4). Therefore, not only the snc genes contribute to sub-exonic circRNA production but also protein-coding genes, lncRNAs and pseudogenes (see below).

No antisense sub-exonic circRNA was detected in porcine and ovine datasets, but we observed that four misc-RNA and two ribozyme genes produced sense and antisense sub-exonic circRNAs in bovine liver and/or testis. Antisense sub-exonic circRNAs were never seen without the corresponding (from the same exon) sense sub-exonic circRNAs.

### Tissue Complexity

#### Analysis of the Number of circRNAs Characterized

We now turn to the comparison of the number of circRNAs observed in liver, muscle and testis in the three species. The circRNAs detected for each dataset by CD, number and associated annotations, exhibit a much higher homogeneity within batches than between batches, indicating that technical bias (library preparation for example) might drive, in part, the observed difference between tissues and species (Figure 3). We noted for example large differences for sub-exonic, intronic and exonic circRNAs number between two batches from porcine liver (ssc_liver_8-10 and ssc_liver_5-7) and two batches from bovine liver (bta_liver_1-12 and bta_liver_13-15) (Figure 4). We noted also large differences of patterns between the two batches from porcine testis (ssc_testis_2-7 and ssc_testis_8-10), but in this case the different age of the animals might drive the differences. Once again, in the CD analysis the number of exonic circRNAs in testis of pubertal animals appeared similar in bovine (bta_testis_4-6) and in pigs (ssc_testis_2-7) (Figures 1G, 4C), and in testis of adult animals in ovine (oar_testis_1-3) and in pigs (ssc_testis_8-10). Nevertheless, we noted that these datasets were generated by different sequencing labs.

In order to avoid the potential technical bias mentioned above, we compared batches originating from the same SRA project. A significant difference was detected in the number of exonic circRNA and in the number of CCRs associated with exonic circRNAs between young and adult animal testes (*p* = 0.016 and *p* = 0.011). These results confirmed data already observed with circRNAs detected by CE2 + CIRI2 (*p* = 0.016). In cattle, (2 × 12 datasets produced at FBN) and in sheep (2 × 6 datasets) we were able to show that the liver transcriptome contains more exonic circRNAs than muscle. The three comparisons (number of exonic circRNAs detected by CD, number of CCRs associated with exonic circRNAs detected by CD and number of exonic circRNAs detected by CE2 + CIRI2) were statistically significant in cattle (*p* = 3E-9, *p* = 3E-9, and *p* = 3E-9, respectively) and in sheep (*p* = 0.0062, *p* = 0.019, and *p* = 0.0031). In contrast, we were not able to confirm this difference in pig. All statistical analyses were reported in Supplementary File 3.

#### Analysis of circRNAs Remaining Without Annotation

Among unannotated circRNAs, a large fraction of very small circRNAs was detected especially in some datasets. For example, in bta_muscle_1-12 we noted 19 to 32% of circRNAs with a genomic size less than 55 bp. It would be necessary to examine the underlying reads to understand this small size, and it is possible that they are false positives. Thus, they were kept as non-annotated circRNAs.

We noticed in porcine muscle that a large proportion of circRNAs was not assigned to a specific chromosome, but was localized on unassigned scaffolds. In ssc_muscle_3 and _4, more than 60% of the circRNAs were localized on these unassigned scaffolds, while only 4.5% of all annotated porcine genes are localized there. For example, in ssc_muscle_3 we counted 3,447 circRNAs localized on these unassigned sequences among the total of 5,540 circRNAs characterized. Even more specifically, 2,494 of them were localized on AEMK02000489 and 930 on AEMK02000695. Except a few sub-exonic circRNAs from a gene, which is probably not fully included in the AEMK02000489 scaffold, these circRNAs remained without annotation. These two scaffolds include only 51 and 16 kb of sequence, respectively, and several RNA genes were suspected to be localized there. These observations about circRNAs assigned to these two scaffolds confirm that they included sequences that are transcribed in muscle. Nevertheless, it cannot be excluded that these are only linear transcripts associated with a bad assembly of the sequences in these scaffolds.

More generally, we searched for circRNA clusters without annotation along the chromosomes, because we suspected that the analysis of clusters of unannotated circRNAs would allow us to highlight regions with sequence/assembly/annotation problems. The first example were 450 circRNAs in bta_muscle_6 (40% of unannotated circRNAs characterized in this dataset) detected in the region BTA-2:18,1-18,3Mb. In ssc_muscle_2, we noted 350 circRNAs without annotation in the region SSC-15:84,23-84,49 Mb. The respective genomic sections contain the *Titin* gene (*TTN*), which is an exceptional gene with probably more than 350 exons spread over 300 kb (data from the Ensembl annotation of the human genome), while only 7 or 13 exons were identified in the TTN annotation of the pig and cattle genomes (Ensembl v-102), respectively. In the sheep genome, the *TTN* gene is not annotated in Ensembl (v-102), but in the dataset oar_muscle_2, 200 circRNA without annotation were detected (30% of unannotated circRNAs characterized in this dataset) in the region suspected to contain this gene. The second example was initiated by the characterization of a cluster of circRNAs without annotation in several bovine liver and muscle datasets. This region on BTA-27: 6.21-6.23Mb is known to contain the *Defensin* gene cluster, which is extremely expanded in ruminants. The assembly in this genomic region is difficult due to a substantial number of copies of the same or very similar sequences. In addition, it is assumed that bovine individuals differ in the number of *Defensin* gene copies. In sheep, this region is apparently not included in the reference genome considered.

There are also regions, where not the genome assembly, but only the annotation is deficient and the impact on the number of detected circRNAs remaining without annotation is very limited. For example in a region of BTA-7 (2,73-2,74 Mb) seven circRNAs without an annotation were detected in bta_muscle_6. NCBI and Ensembl do not annotate a gene there. However, RNA-seq data displayed at NCBI would strongly support a gene annotation and a new gene with a very large number of exons had been annotated (Nolte et al., 2020).

To finish, a last example, one circRNA was detected only in the dataset ssc_muscle_4 but with a very high number of CCRs by CD (and with a very high number of BSJs by CIRI2). This circRNAs could be explained by a fusion between one exon from ENSSSCG00000029441 (probably *MYH2*) and one from ENSSSCG00000018005 (*MYH8*). A potential fusion of exons from two annotated genes would explain why the annotation-dependent CE2 pipeline did not retain this circRNA. Even though we cannot discard the hypothesis of a deficiency in genome annotation, we believe rather this to be a structural alteration in the respective genomic region restricted to this particular animal.

The above examples underline that the accuracy of the reference assembly and of the annotation has a major impact on the number of unannotated circRNAs preventing from drawing any conclusion from the observed difference between tissues and samples.

#### Intronic circRNAs

In bovine and porcine datasets, respectively 53 introns (from 53 genes) and 80 introns (from 79 genes) were able to produce intronic circRNAs. A large part of intronic circRNAs characterized in bovine and porcine datasets were mainly detected in testicular datasets. In porcine datasets, the top-ranked expressed intronic circRNA, from an intron of *ATXN2L*, has ten times more CCRs than the second ranked one (*PEX10*). This intronic circRNA was detected in each of the 18 porcine datasets and is always among the strongest contributors of intronic circRNAs. Even though *ATXN2L* was ranked at the second position in terms of CCRs associated with intronic cirRNAs in bovine testis, the landscape of the production of intronic circRNAs in bovine testis is not dominated by the production of a particular intron. The *ATXN2L* intron concerned is in an orthologous position in pig and cattle. The orthologous ovine gene is not annotated in the reference genome used (Oar_rambouillet_v1.0).

The number of intronic circRNA (Figure 4B) seems to be related to the number of exonic circRNA (Figure 4A) but not to the number of sub-exonic circRNAs (Figure 4C). Specifically, the number of CCRs associated to intronic circRNAs appears to be correlated to the number of CCRs associated to exonic circRNA in porcine and in bovine testis (Figures 4D,E). If we consider the six bovine testicular datasets (from young and pubertal animals), the correlation coefficient is 0.92. The correlation is similar between the six porcine testicular datasets from pubertal animals regardless of whether all introns with intronic circRNAs are considered (Figure 4D) or if the *ATXN2L* intron with the highest intronic circRNA expression is excluded (*r* = 0.88) (Figure 4E). Excluding CCRs from *ATXN2L* enables a comparison of intronic circRNA contents for bovine and porcine testis at the same scale.

So far, intronic circRNAs have been identified only from coding genes (reviewed by Robic and Kühn, 2020), but the current study shows that lncRNAs can also be involved in the production of intronic circRNAs. In pigs, this study highlighted that the lncRNAs ENSSSCG00000048463 and ENSSSCG00000041596 generated intronic circRNAs, however at a low expression level, with 6 and 11 associated CCRs, respectively.

#### Analysis of the Production of Sub-Exonic circRNAs

To analyze the catalog of genes capable of producing sub-exonic and/or exonic circular RNAs, we examined the 2 × 12 datasets produced at FBN from bovine liver and muscle. We observe that 1,914 and 839 genes are able to produce exonic circRNAs in liver and muscle, respectively, while only 472 and 228 are able to produce sub-exonic circRNAs in the respective tissues. Only 124 genes produce both exonic and sub-exonic circRNAs in liver, while in muscle we find only 37. The ability to produce sub-exonic circRNA is therefore not related to the ability to produce exonic circRNA.

The top-3 ranked genes producing sub-exonic circRNAs in bovine liver are *ALB, COX1 and FGB*. In bovine muscle, we could identify *COX1, MYH1, MYH2*, and *ACTA1* among the top-5 ranked genes producing sub-exonic circRNAs. In ovine muscle, *XIRP2*, *MYH1*, *ACTA1*, and *MYH7* are among the top-6 ranked genes. Two myosin genes are found in the top ranking list of the strongest contributors to sub-exonic circRNAs in porcine muscle. In ovine liver, nearly half of the CCRs are assigned to sub-exonic circRNAs produced by *ALB*. In the porcine liver, *ALB, FGB*, and *FGA* are the top-3 genes producing sub-exonic circRNAs. In porcine and ovine testis, the strongest contributor of sub-exonic circRNAs is *HSPCA.* All these coding parent genes producing a large number of sub-exonic circRNA are also known to be among the top-ranked contributors of linear transcripts in the respective tissue. The contribution of protein-coding genes to the production of sub-exonic circRNAs represents a large fraction of CCRs characterizing sub-exonic circRNAs (especially in bovine liver and muscle). Moreover, the list of protein coding genes providing the strongest contribution of sub-exonic circRNA seems to be a direct reflection of the respective list for linear transcript contribution. This would be a feature of sub-exonic circRNAs that is not shared with exonic circRNA.

In previous studies, sub-exonic circRNAs had been searched in mono-exonic nc genes (Robic et al., 2020). However, also coding mono-exonic genes contribute to sub-exonic circRNA production. Specifically, the gene *COX1* is a mono-exonic gene localized on the mitochondrial genome. It is among genes able to produce a high number of sub-exonic circRNAs in bovine and ovine liver and in bovine and ovine muscle, while in pigs its contribution is insignificant (in liver and muscle). In cattle, where 13 protein-coding genes are described on the mitochondrial genome, 12 were identified as able to produce sub-exonic circRNAs. All these 13 mitochondrial protein-coding genes are mono-exonic genes (Taanman, 1999).

In spite of our new data on the contribution of coding genes to circRNA production, non-coding genes were also important contributors of sub-exonic circRNA in some datasets. *RNase_MRP* is the strongest contributor of sub-exonic circRNA in each dataset of ssc_testis_2-7. This observation confirms data obtained previously on ssc_testis_1 (Robic et al., 2020). Among the other 60 datasets, there is an important contribution of *RNase_MRP* to sub-exonic circRNAs in oar_liver_5. We also noted a significant contribution of *RNaseP_nuc* to subexonic circular RNAs in bta_muscle_9 while no sub-exonic circular RNA of this gene was detected in the other bovine muscle datasets. Nevertheless, apart from the sub-exonic circRNAs produced by ribozymes, we have to be careful with respect to the possible production of sub-exonic circRNAs by sncRNAs. Some batches seem to be very rich in some sncRNAs while others display not a single read aligned on the respective reference genome (data not shown). With the currently available data and metadata descriptions, it is difficult to differentiate a tissue/age specificity from a difference resulting from a technical bias e.g., the RNA extraction methodology.

Our data show that genes able to produce sub-exonic circRNA can be separated into two sub-groups. Mono-exonic and nc genes were already described as being able to produce sub-exonic circRNAs, and this study shows that mitochondrial genes, which are protein-coding and mono-exonic genes, are also concerned. Furthermore, this study demonstrates that multi-exonic genes, in particular protein-coding genes, can also produce sub-exonic circRNAs. In addition, the coding-genes that are major contributors of sub-exonic circRNAs strongly contribute also to the production of linear transcripts. All data were reported in Supplementary List 4.

#### circRNAs and Non-coding Genes

The current knowledge about nc genes is still poor in the livestock species investigated here, which has an impact on evaluating their contribution to circRNA production. While there is a similar number of protein-coding genes annotated in livestock and human genomes (cow-21,861, pig-21,280, sheep-20,477, human-20,448, Ensembl v-101), comparing the number of annotated pseudogenes (cow-492, pig-1,626, sheep-830, human-15,217) and of lncRNAs (cow-1,480, pig-6,790, sheep-2,229, human-16,909) demonstrates that non-coding genes are still poorly described in livestock species. This study shows that nc genes can also contribute to the production of intronic, sub-exonic and exonic circRNAs. For the production of intronic circRNAs, the current study highlights only two lncRNAs, but for the sub-exonic circRNAs, the contribution of non-coding genes is unquestionable (Table 2). In cattle and pigs, 945 and 998 genes were characterized as able to produce sub-exonic circRNAs (Table 2), respectively. Among them, 4 and 18 were lncRNAs. Surprisingly in sheep, where only 462 genes are characterized as able to produce sub-exonic circRNAs, we found a relatively higher number of lncRNAs (15). There is a much higher number of lncRNAs involved in exonic circRNA production in sheep compared to pigs considering the number of overall annotated lncRNAs (CE2 + CIRI2, Table 3). For example, in ovine testis, we observed that the strongest contributor of exonic circRNAs in terms of BSJ (Supplementary Table 2) is a lnc. This lncRNA was able to produce eleven exonic circRNAs of which the three most expressed were ranked at #2, #38, and #50 among the top-ranked exonic circRNAs in ovine testis (Supplementary Table 1). This might be due to fewer, but more precisely annotated lncRNA genes in sheep including a higher number of described exons. All non-coding genes, which were confirmed by both approaches (CE2 + CIRI2 and CD) as able to produce exonic circRNAs, are reported in Supplementary File 4. Non-coding genes are also involved in the production of exonic circRNA, not only lncRNAs but also pseudogenes and snoRNAs (Table 3).

**TABLE 2:** Coding and non-coding genes are to produce sub-exonic circRNAs.

Genes concerned by sub-exonic circRNAs		Protein-coding genes		Long non-coding genes		Pseudogenes		Small non-coding genes
		N. of genes concerned	N. of exons concerned	N. of genes concerned	N. of exons concerned	N. of genes concerned	N. of exons concerned	N. of genes concerned
Cattle	945	908	1,451	4	4	3	3	30
								11 misc_RNA, 2 ribozyme, 2 Snc, 2 Sca_RNA, 13 Sno
Pig	998	954	1,228	19	22	6	7	19
								2 misc_RNA, 2 ribozyme, 2 Sca_RNA, 12 Sno, 1 Y_RNA
Sheep	465	434	569	15	20	7	9	9
								1 misc_RNA, 2 ribozyme, 1 Sca_RNA, 5 Sno

*Genes able to produce sub-exonic circRNAs were characterized using CD approach. Lists were available in Supplementary List 4.*

**TABLE 3:** Non-coding genes able to produce exonic circRNAs.

	lncRNAs No. of genes concerned	Pseudogenes No. of genes concerned	sncRNAs No. of genes concerned
Cattle	6	0	1 Sno
Pig	32	7	1 Sno
Sheep	103	2	0

*Only non-coding genes highlighted by both approaches used for circRNA detection (CE2 + CIRI2 and CD) were considered as able to produce exonic circRNAs. Their respective Ensembl_Id were reported in Supplementary File 4.*

### Comparison of Circular Transcriptomes (exonic circRNAs)

#### Comparison of Circular Transcriptomes Between Tissues

When we examined the number of genes producing the 100 strongest expressed exonic circRNAs (CE2 + CIRI2) of a given batch (hereafter Top-100/circRNAs), we counted 87 to 89 distinct genes for the five porcine batches, and 92 to 93 for the three ovine batches. For four bovine batches 90 to 97 genes produced the 100 strongest expressed exonic circRNAs, the exception was in muscle where only 83 distinct parent genes were identified (Supplementary Table 1). As we previously proposed a comparison based on the most highly expressed exonic circRNA (Figure 2A), we propose now a comparison based on genes with the highest expression in terms of BSJ. The Top-100 list of parent genes most strongly producing exonic circRNAs was established for each batch from the three species (hereafter Top-100/genes, Supplementary Table 2), and analyzed. The levels of overlap between testis/liver/muscle appeared higher in sheep than in cattle or pigs (Figure 2B-3 in yellow). In cattle, the levels of overlap observed for pairwise comparisons of Top-100/genes (Figure 2B-1) were similar to those noted for Top-100/circRNAs (Figure 2A-1 in blue). The main exception is the overlaps between the two batches from bovine liver (87% for Top-100/genes and 72% for Top-100/circRNAs). In pigs, the level of overlap observed for pairwise comparisons of Top-100/genes (Figure 2B-2) were almost systematically higher than those noted for Top-100/circRNAs (Figure 2A-2). On Top-100/genes, overlaps between the pubertal and adult batches from porcine testis (84%) were better than the overlaps observed between the two batches from porcine liver (74% for Top-100/genes, Figure 2B-2). Exonic circRNA production with high BSJ seemed focused on a group of genes that produce several distinct exonic circRNAs with a balance of circular isoforms which would be dependent on considered tissue (Supplementary Table 1 and Supplementary File 5). One example to illustrate this: The gene *NUP210L* was ranked at the second position in terms of BSJs counts in porcine pubertal testis, and 30 distinct exonic circRNAs were characterized with a dominant form. In adult porcine testis this gene was ranked at the third position with 23 circular isoforms characterized but without dominant form. When we examined the Top-100/genes established for the porcine batches (Supplementary Table 2), we noticed that *KANSL1L* was among the strongest producers of exonic circRNAs (CE2 + CIRI2) in the five batches (#1 to #7). The bovine orthologous gene produced also many exonic circRNAs, with the highest ranking noted for testis from young bulls (#17) and the lowest ranking for pubertal testis (#62).

#### Impact of Genes Able to Produce Multiple Exonic circRNAs

We further examined genes with multiple exonic circRNAs characterized. The protein-coding gene *SMARCC1* is able to produce 41 distinct exonic circRNAs in the porcine testis (CE2 + CIRI2) (Supplementary File 5). Less than 30 exons were reported for this porcine gene (as in humans), which are spread across 190 kb (Ensembl, v-102). In contrast, the bovine *SMARCC1* is able to produce only five distinct exonic circRNAs in testis and no circRNAs from the ovine *SMARCC1* gene were identified. This result was confirmed among the exonic cirRNAs characterized by CD. The strongest producer of distinct circRNAs (Supplementary File 5) in bovine testis is *DNAH14*, but this gene does not appear in the ovine and porcine lists, because of poor knowledge (sequences/annotation) about this gene in these species. In muscle, the gene producing the largest diversity of circRNAs is the same in the three species (Supplementary File 5). The *Nebulin* gene (*NEB*) is a gene with a large number of exons. In humans, more than 180 exons were characterized in a region of 200 kb (Ensembl v-102). Among the three species investigated in our study, the highest number of distinct exonic circRNAs from this gene is noted for cattle. In the three species, *NEB* produced this diversity of exonic circRNAs quasi-exclusively in muscle (29/30 cattle, 15/16 pigs, 17/19 sheep). When we examined lists of genes present in Top-100/genes (Supplementary Table 2), we noticed that these lists contained mainly genes with multiple exonic circRNAs characterized. *NEB* was a good example to illustrate this. In porcine and ovine muscle, *NEB* was ranked at the third position of genes expressing a high quantity of circular transcripts where 16 and 19 circular isoforms were respectively characterized (Supplementary Table 2). In pigs and in sheep, a circular form among all exonic circRNAs from *NEB* appeared dominant and this dominant form was ranked at #2 and #4 on the ovine and porcine Top-100/circRNAs in muscle (Supplementary Table 1). These dominant alternative circular transcripts are not pig-sheep orthologous circRNAs. In bovine muscle, 30 distinct *NEB* circRNAs were characterized and this parent gene was ranked at the second position in terms of BSJs counts (Supplementary Table 2). Even though the expression of the strongest expressed alternative form of *NEB* circRNA was 20 times higher than the lowest, there was not really a dominant form in bovine muscle.

#### Comparison of the Circular Transcriptome Between Species

**FIGURE 5:**
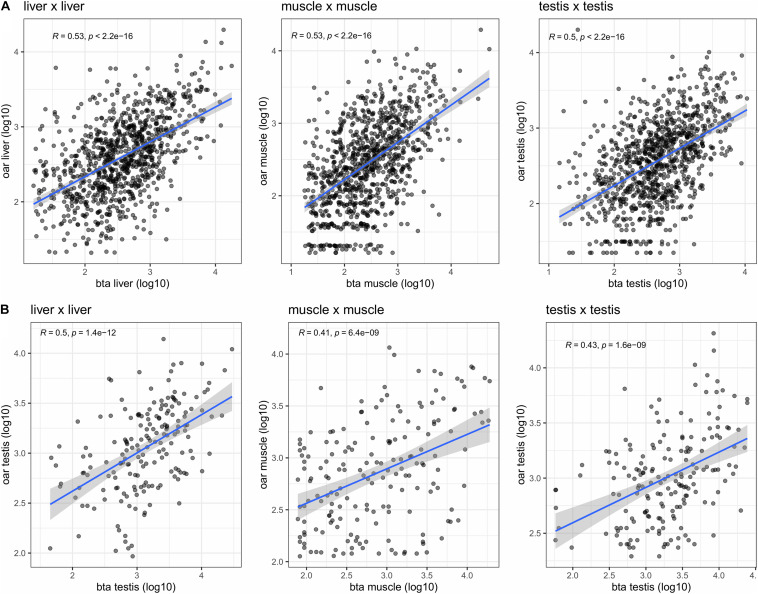
Comparative analysis of the bovine and ovine transcriptomes. **(A)** Circular expression profiles of orthologous genes (measured as number of BSJs) were compared in liver, and in muscle. **(B)** Expression profiles of orthologous circRNAs (measured as number of BSJs) were compared in liver, and in muscle. Only genes/circRNAs with a substantial expression were kept for these correlation analyses (log10(BSJs) > 1.2 for each tissue and each species).

The comparison of the expression profiles between species was performed using two different approaches. First, a comparison between the expression profiles of orthologous parent genes was performed (see Methods). We observe here a clear correlation between the expression profile between species for the same tissue as exemplified by the comparison of sheep and bovine tissues expression profiles (Figure 5A). Ranking the expression profiles from highest to lowest for each species and each tissue however underlines that this ranking is not strictly conserved between the three species (Table 4). From the examination of this table, it would be tempting to deduce (Table 4) that for example (1) the circular transcripts expression of ovine *SUGT1* would be a differential characteristic with respect to pig muscle (2) the circular transcripts expression of ovine *TRDN* would be a differential characteristic with respect to bovine muscle. When we examined respective annotations available, this suggestion appeared possible for *SUGT1*. In contrast, this suggestion did not stand up to the examination of the annotation of the bovine *TRDN* gene. Moreover, when we examined these data, we found no clear overlap as demonstrated by two examples: (1) The *NEB* gene was ranked high across all three species: at #3 on the ovine list and also #3 on the porcine list and #23 on the bovine list. (2) In contrast, the #8 of the ovine list (*SLC9A2*) was found at #60 and #2,854 on the bovine and porcine lists, respectively. The genes that are the strongest producers of circRNA in sheep muscle are not necessarily genes that produce a lot of circRNA in pig or cattle muscle.

**TABLE 4:** Strongest parent genes for cirRNA production identified in muscle.

Ovine gene	Rank in oar_muscle	Bovine orthologous gene	Rank in bta_muscle	Porcine orthologous gene	Rank in ssc_muscle
ENSOARG00020005060 *SUGT1*	1	ENSBTAG00000002137	382	ENSSSCG00000039338	–
ENSOARG00020003405 *UBE3A*	2	ENSBTAG00000002487	1,005	ENSSSCG00000004832	4
ENSOARG00020024247 *NEB*	3	ENSBTAG00000006907	23	ENSSSCG00000016397	3
ENSOARG00020019783 *ANO5*	4	ENSBTAG00000019394	392	ENSSSCG00000013344	1,673
ENSOARG00020010566 *LMO7*	5	ENSBTAG00000010693	2	ENSSSCG00000040184	386
ENSOARG00020016807 *PPP2R3A*	6	ENSBTAG00000023416	233	ENSSSCG00000033185	1,189
ENSOARG00020020875 *TRDN*	7	ENSBTAG00000038849	–	ENSSSCG00000027613	263
ENSOARG00020002529 *SLC9A2*	8	ENSBTAG00000001706	60	ENSSSCG00000008153	2,854
ENSOARG00020012532 *SENP6*	9	ENSBTAG00000005869	1	ENSSSCG00000020702	733
ENSOARG00020003993 *ANO6*	10	ENSBTAG00000002902	130	ENSSSCG00000000804	–
ENSOARG00020021163 *SLTM*	11	ENSBTAG00000011319	853	ENSSSCG00000004592	20
ENSOARG00020002353 *SNX13*	12	ENSBTAG00000014074	207	ENSSSCG00000024761	273
ENSOARG00020006228 *MYBPC1*	13	ENSBTAG00000011392	3	ENSSSCG00005042452	–
ENSOARG00020025783 *GOLGA4*	14	ENSBTAG00000016563	377	ENSSSCG00000011243	–
ENSOARG00020017442 *ZEB1*	15	ENSBTAG00000020053	704	ENSSSCG00000011025	630

*At left, the list is provided encompassing the top-15 parent genes for exonic circular RNAs with respect to the number of BSJs identified in sheep muscle. In the center and on the right, the respective ranking is given for orthologous genes in the list of parent genes for exonic circular RNA in bovine and porcine muscle.*

In order to bypass the limitation of comparing the expression of parent genes, we identified directly orthologous circRNAs based on sequence similarities (see Methods). From 3,899 ovine circRNAs and 8,723 bovine circRNAs, we were able to identify 1,832 orthologous circRNAs (see Suppl_Lists-1). For comparison, the Ensembl one-to-one ortholog set contains 16,110 orthologs, among 21,861 bovine and 20,477 ovine coding genes. Again, we observe a correlation between the expressions of circRNAs between species for the same tissue (Figure 5B) suggesting that, just as the linear transcriptome (Kryuchkova-Mostacci and Robinson-Rechavi, 2016), the circRNA transcription profile is, at least in part, conserved across species.

## Discussion

The imbalance between batches observed during the circRNA characterization phase and other issues (e.g., incomplete genome assemblies/annotations) precluded us to perform a global comparison between tissues and between species. Instead, we highlight a few examples to show different and sometimes seemingly contradictory results to demonstrate the complex issue of these comparisons.

Even though each of the datasets considered individually was of correct quality, the agglomeration of datasets from different origins proved to be difficult. This was a surprising outcome, because many studies used a similar approach with mRNA-seq datasets from different sources (Soumillon et al., 2013; Fang et al., 2020). However, while it is difficult to compare the number of circRNAs when datasets come from different sources, the contents in circRNAs (the most expressed circRNAs or the genes with the highest expressing of circRNA in terms of BSJ) are quite comparable. We can put up the hypothesis that protocols for RNA preparation and sequencing have a significant impact on circRNA recovery. We assume that a subset of circular RNAs may be present in tissues in a complex form and that RNA purification methods may differ with respect to their recovery (Pamudurti et al., 2017; Ragan et al., 2019). The differences between RNA preparation protocols are not always well explained but, for example, we are sure that there are differences in the use or non-use of TRIzol, which exist between batches for this study. For example, the RNA produced for the ssc_testis_1-7 datasets was obtained from a dry-powdered tissue sample before being treated with TRIzol (Robic et al., 2016). In contrast, datasets generated at FBN or at Roslin Institute were produced from a tissue sample homogenized directly in TRIzol (Clark et al., 2017; Nolte et al., 2019). Moreover, an additional on-column-purification step was performed for RNAs extracted at FBN (bta_muscle_1-12) (Nolte et al., 2019) or at INRAE (ssc_testis_1-7) (Robic et al., 2019). Some protocols are described too succinctly to be sure that this type of additional step did not performed. These observations demonstrate the need for harmonized or at least fully described laboratory methods attached as metadata to enable samples to be fully useful in functional annotation of genomes as agreed upon in the global FAANG initiative.

Although the diversity regarding the source of datasets had somewhat limited our analyses, we were able to show that the ruminant liver contains more exonic circRNA than muscle. In testis, the number of exonic circRNAs seemed associated with the age of the animals. In bulls, the testis contained more circRNAs at birth than at puberty. An inverse dynamic was observed in rat (Zhou et al., 2018; Gong et al., 2020). Nevertheless, at birth, a rodent’s testis presents large differences with a bull testis (Fujihara et al., 2011; McGowan et al., 2018; Picut et al., 2018). When we compared the circRNA expression of two datasets from the same tissue of the same species, we observed differences, but much less than those between two tissues of the same species. However, the similarities between species are more difficult to quantify, because annotations relative to parent genes are often deficient in at least one of the species considered. The overlap between pubertal testis and testis at other stages led to an intermediate value and showed that the testis is a tissue with a maturation in progress. We showed that there are points of agreement in the circular transcriptome of the same tissue in two species, but also many divergences. Some of the strongest parental genes for exonic circular RNAs may be also among those genes, which produce a large quantity of circular transcripts in several tissues. Nevertheless, this characteristic of high circRNA expression across tissues may be limited to one species (*KANSL1L* in pigs). Moreover, the parent genes of exonic circRNAs are often capable of producing several distinct circRNAs. This has an impact on the composition of the circular transcriptome and the balance between the different circular isoforms contributed also to the composition of circular transcriptome. It seems that among these circular isoforms, there may be a dominant form, but this is not a rule. The balance between the different isoforms will have to be studied in the future, as this seems to be a very specific question for the circular transcriptome. Analyses presented here showed that it is not enough to have a set of orthologous genes capable of producing circRNAs to obtain a similar circRNA landscape in the same tissue from both species. The fact that exonic circRNAs can be produced from the same exons (orthologous circRNA as found by Suenkel et al., 2020) or not appears at this stage as a detail. For example *SMARCA5* is known to produce exonic circRNAs in connection with biological data in humans (Kong et al., 2017) and pigs (Robic et al., 2019), but the exons involved are different. One future direction might be to take into account the relative “weight” of exonic circRNAs/parent genes for comparative analyses of several circular transcriptomes. Among lists of genes able to produce exonic circRNAs in one species, we can find a proportion of genes, which are able to produce exonic circRNAs also in a second species and in the same tissue. Nevertheless, the relative contribution of those genes to the second circular transcriptome is not predictable. There may be points of concordance in the circular transcriptome of the same tissue in two species, but it will be difficult to conclude from one species to another demonstrating the need to conduct a comprehensive characterization within each species. Besides, we would like to emphasize that in this study we considered circRNAs distinguishable by their circular junction. We know nothing about the internal structure of circRNA and multiple distinct circular RNAs can share the same circular junction (Dodbele et al., 2021).

This study shows that multi-exonic genes can also generate sub-exonic circRNAs. These multi-exonic genes were most often protein-coding genes, but some lncRNA and pseudogenes were also highlighted. Because the list of genes, which are the strongest contributors of sub-exonic circRNAs (especially in bovine liver and muscle), seems to be a direct reflection of the respective list of contributors of linear transcripts, we suspect that these circRNAs from multi-exonic genes are mostly the result of splicing machinery errors or a destruction process of linear transcripts. For sub-exonic circRNA from mono-exonic genes, this current study confirmed the previous study (Robic et al., 2020, which was based only on the ssc_testis_1 dataset) that ribozymes and other snc-RNA genes are able to produce sub-exonic circRNAs. We provide data supporting the production of sub-exonic circRNAs by mitochondrial protein-coding genes, which is new, but not a surprise. The transcription of these mono-exonic genes does not require the splicing step (no intronic sequence to be removed), and frequently sub-exonic circRNAs include a notable part of potentially transcribed sequences (unique exons). These genes have probably kept some features of ancestral genes (prokaryotic genes) including the production of circRNAs (Danan et al., 2012). It was already described for ribozymes (Cervera and de la Pena, 2020). We believe that it is a constitutive phenomenon relative to these mono-exonic genes, where a part of transcripts is circular. We cannot rule out the hypothesis that the transcripts of these genes adopt the circular form for a better life span or a best biological efficiency.

When we wanted to compare results between species, we were confronted with problems related to the reference genome, because some of the genes were not annotated with the same quality in all species. We would like to emphasize that the assignment of a given circRNA to a parent gene is dependent on the knowledge of the genome and available annotation. We began this study with strict constraints on the annotation of the parent circRNA gene (see Materials and Methods). Although excellent analyses to compare circular transcriptomes have recently been published using a different approach (Ji et al., 2019), we still believe that, especially in animal species, it is important to perform comparative studies with only circRNAs with a clearly identified parent gene (Dong et al., 2017). Moreover, this approach avoid also a large number of false positive circRNA annotations (Kaur et al., 2018; Ragan et al., 2019). This study shows that nc genes can give rise to intronic, sub-exonic and exonic circRNAs. For exonic circRNAs, we were surprised to observe the highest similarities between tissues and the smallest number of distinct exonic circRNA in sheep. Our observations on circRNAs showed that the ovine reference genome might suffer from a deficit of described exons in protein-coding genes. The ovine lncRNAs included in the genome annotation, however, seemed to be better described than lncRNAs from pigs. We would underline that the current knowledge about nc genes is still poor in the livestock species investigated here (Gao et al., 2019; Nolte et al., 2020). When we started this study, we assumed that unannotated circRNAs would reveal the existence of new exons/transcripts/genes, and we thought that the list contained many circRNAs that could be annotated with a single effort on genome annotation as suggested in a previous study (Robic et al., 2020). Nevertheless, the current study revealed that the vast majority of unannotated circRNAs were grouped in clusters along the genome (especially in muscle). We showed that these clusters pointed to genomic regions with problems regarding gene annotation/assembly/sequences. In these genomic regions, the problems are often multiple, and the identification of new linear or circular transcripts seems to be a dangerous process, if it is not associated with a real parallel effort on linear transcriptome annotation and even an improved genome assembly.

This study highlights the importance of improving genome annotation to better annotate circRNAs observed. To our disappointment, not all detected circRNAs can directly contribute to the annotation of new genes. Nevertheless, we believe that a “wide-angle” approach to study circular RNAs can help locate genomic regions with multiple problems. This study highlights the importance of improving genome annotation to better understand the circRNA production.

## Data Availability Statement

The original contributions presented in the study are included in the article/Supplementary Material, further inquiries can be directed to the corresponding author/s.

## Author Contributions

AR, TF, and CK conceived the overall idea of the study and analyzed the results. CC built the bioinformatics tool to characterize circRNAs with the assistance of TF. CC and TF performed all bioinformatics analyses. TF coordinates the study and analyses. AR performed the formal analysis, validation, and data curation, and prepared and wrote the original draft. All authors reviewed the manuscript, read and agreed to the published version of the manuscript, and contributed substantially to the work reported.

## Conflict of Interest

The authors declare that the research was conducted in the absence of any commercial or financial relationships that could be construed as a potential conflict of interest.

## Footnotes

1 www.animalgenome.org/community/FAANG/

## Supplementary Material

Supplementary File 1 Some features relative to circRNAs characterization (CE2 + CIRI2).

Supplementary File 2 Characterization of exonic circRNAs performed by CE2 + CIRI2.

Supplementary File 3 All statistics relative to comparisons of the number of circRNAs.

Supplementary File 4 List of non-coding genes considered as able to produce exonic circRNAs (CE2 + CIRI2 and CD).

Supplementary File 5 Genes able to produce multiple exonic circRNAs (CE2 + CIRI2).

Supplementary Table 1 Top-100/circRNAs: Lists of 100 strongest expressed circRNAs in 5 porcine, 5 bovine and 3 ovine batches.

Supplementary Table 2 Top100/parent genes: Lists of 100 genes expressed the most circRNAs (in term of BSJ) in 5 porcine, 5 bovine and 3 ovine batches.

Supplementary List 1 This file consists of 6 large sheets listing the exonic circRNAs detected by CE2 + CIRI2 and also the bovine-ovine orthologous circRNAs.

Supplementary List 2 This file consists of 6 large sheets relative to exonic circRNAs detection by CD and their parent genes.

Supplementary List 3 This file consists of 6 sheets relative to intronic circRNAs detection by CD and their parent genes.

Supplementary List 4 This file consists of 9 large sheets relative to sub-exonic circRNAs detection by CD and their parent genes.

## References

[B1] Au C. H. Wa A. Ho D. N. Chan T. L. Ma E. S. (2016). Clinical evaluation of panel testing by next-generation sequencing (NGS) for gene mutations in myeloid neoplasms. *Diagn. Pathol.* 11:11. 10.1186/s13000-016-0456-8 PMC4722624 26796102

[B2] Barrett S. P. Parker K. R. Horn C. Mata M. Salzman J. (2017). ciRS-7 exonic sequence is embedded in a long non-coding RNA locus. *PLoS Genet.* 13:e1007114. 10.1371/journal.pgen.1007114 29236709 PMC5745005

[B3] Cattle (2021). *Cattle.* Cambridge: Ensembl. http://www.ensembl.org/Bos_taurus/Info/Index

[B4] Cervera A. de la Pena M. (2020). Small circRNAs with self-cleaving ribozymes are highly expressed in diverse metazoan transcriptomes. *Nucleic Acids Res.* 48 5054–5064. 10.1093/nar/gkaa187 32198887 PMC7229834

[B5] Chen L. L. (2020). The expanding regulatory mechanisms and cellular functions of circular RNAs. *Nat. Rev. Mol. Cell Biol.* 21 475–490. 10.1038/s41580-020-0243-y 32366901

[B6] Chen L. Yang R. Kwan T. Tang C. Watt S. Zhang Y. (2020). Paired rRNA-depleted and polyA-selected RNA sequencing data and supporting multi-omics data from human T cells. *Sci. Data* 7:376. 10.1038/s41597-020-00719-4 PMC7652884 33168820

[B7] Cheng J. Metge F. Dieterich C. (2016). Specific identification and quantification of circular RNAs from sequencing data. *Bioinformatics* 32 1094–1096. 10.1093/bioinformatics/btv656 26556385

[B8] Clark E. L. Bush S. J. McCulloch M. E. B. Farquhar I. L. Young R. Lefevre L. (2017). A high resolution atlas of gene expression in the domestic sheep (Ovis aries). *PLoS Genet.* 13:e1006997. 10.1371/journal.pgen.1006997 28915238 PMC5626511

[B9] Danan M. Schwartz S. Edelheit S. Sorek R. (2012). Transcriptome-wide discovery of circular RNAs in Archaea. *Nucleic Acids Res.* 40 3131–3142. 10.1093/nar/gkr1009 22140119 PMC3326292

[B10] Das D. Das A. Sahu M. Mishra S. S. Khan S. Bejugam P. R. (2020). Identification and Characterization of Circular Intronic RNAs Derived from Insulin Gene. *Int. J. Mol. Sci.* 21:4302. 10.3390/ijms21124302 32560282 PMC7352490

[B11] Dobin A. Davis C. A. Schlesinger F. Drenkow J. Zaleski C. Jha S. (2013). STAR: ultrafast universal RNA-seq aligner. *Bioinformatics* 29 15–21. 10.1093/bioinformatics/bts635 23104886 PMC3530905

[B12] Dodbele S. Mutlu N. Wilusz J. E. (2021). Best practices to ensure robust investigation of circular RNAs: pitfalls and tips. *EMBO Rep.* 22:e52072. 10.15252/embr.202052072 PMC7926241 33629517

[B13] Dong R. Ma X. K. Chen L. L. Yang L. (2017). Increased complexity of circRNA expression during species evolution. *RNA Biol.* 14 1064–1074. 10.1080/15476286.2016.1269999 27982734 PMC5680680

[B14] Fang L. Cai W. Liu S. Canela-Xandri O. Gao Y. Jiang J. (2020). Comprehensive analyses of 723 transcriptomes enhance genetic and biological interpretations for complex traits in cattle. *Genome Res.* 30 790–801. 10.1101/gr.250704.119 32424068 PMC7263193

[B15] Fujihara M. Kim S. M. Minami N. Yamada M. Imai H. (2011). Characterization and in vitro culture of male germ cells from developing bovine testis. *J. Reprod. Dev.* 57 355–364. 10.1262/jrd.10-185m 21289464

[B16] Gao Y. Zhao F. (2018). Computational Strategies for Exploring Circular RNAs. *Trends Genet.* 34 389–400. 10.1016/j.tig.2017.12.016 29338875

[B17] Gao Y. Li S. Lai Z. Zhou Z. Wu F. Huang Y. (2019). Analysis of Long Non-Coding RNA and mRNA Expression Profiling in Immature and Mature Bovine (*Bos taurus*) Testes. *Front. Genet.* 10:646. 10.3389/fgene.2019.00646 31333723 PMC6624472

[B18] Gao Y. Wang J. Zhao F. (2015). CIRI: an efficient and unbiased algorithm for de novo circular RNA identification. *Genome Biol.* 16:4. 10.1186/s13059-014-0571-3 PMC4316645 25583365

[B19] Gao Y. Zhang J. Zhao F. (2018). Circular RNA identification based on multiple seed matching. *Brief Bioinform.* 19 803–810. 10.1093/bib/bbx014 28334140

[B20] Gong B. Xu J. Tong W. (2020). Landscape of circRNAs Across 11 Organs and 4 Ages in Fischer 344 Rats. *Chem. Res. Toxicol.* 34 240–246. 10.1021/acs.chemrestox.0c00144 32692164

[B21] Hansen T. B. (2018). Improved circRNA Identification by Combining Prediction Algorithms. *Front. Cell Dev. Biol.* 6:20. 10.3389/fcell.2018.00020 29556495 PMC5844931

[B22] Hansen T. B. Jensen T. I. Clausen B. H. Bramsen J. B. Finsen B. Damgaard C. K. (2013). Natural RNA circles function as efficient microRNA sponges. *Nature* 495 384–388. 10.1038/nature11993 23446346

[B23] Jeck W. R. Sorrentino J. A. Wang K. Slevin M. K. Burd C. E. Liu J. (2013). Circular RNAs are abundant, conserved, and associated with ALU repeats. *RNA* 19 141–157. 10.1261/rna.035667.112 23249747 PMC3543092

[B24] Ji P. Wu W. Chen S. Zheng Y. Zhou L. Zhang J. (2019). Expanded Expression Landscape and Prioritization of Circular RNAs in Mammals. *Cell Rep.* 26 3444–3460e3445. 30893614 10.1016/j.celrep.2019.02.078

[B25] Kaur S. Mirza A. H. Pociot F. (2018). Cell Type-Selective Expression of Circular RNAs in Human Pancreatic Islets. *Noncoding RNA* 4:38. 10.3390/ncrna4040038 30486482 PMC6316812

[B26] Kong Z. Wan X. Zhang Y. Zhang P. Zhang Y. Zhang X. (2017). Androgen-responsive circular RNA circSMARCA5 is up-regulated and promotes cell proliferation in prostate cancer. *Biochem. Biophys. Res. Commun.* 493 1217–1223. 10.1016/j.bbrc.2017.07.162 28765045

[B27] Kristensen L. S. Andersen M. S. Stagsted L. V. W. Ebbesen K. K. Hansen T. B. Kjems J. (2019). The biogenesis, biology and characterization of circular RNAs. *Nat. Rev. Genet.* 20 675–691. 31395983 10.1038/s41576-019-0158-7

[B28] Kryuchkova-Mostacci N. Robinson-Rechavi M. (2016). Tissue-Specificity of Gene Expression Diverges Slowly between Orthologs, and Rapidly between Paralogs. *PLoS Comput. Biol.* 12:e1005274. 10.1371/journal.pcbi.1005274 28030541 PMC5193323

[B29] Li H. M. Ma X. L. Li H. G. (2019). Intriguing circles: Conflicts and controversies in circular RNA research. *RNA* 10:e1538. 10.1002/wrna.1538 31034768

[B30] Liu X. Hu Z. Zhou J. Tian C. Tian G. He M. (2020). Interior circular RNA. *RNA Biol.* 17 87–97. 10.1080/15476286.2019.1669391 31532701 PMC6948956

[B31] McGowan M. Holland M. K. Boe-Hansen G. (2018). Review: Ontology and endocrinology of the reproductive system of bulls from fetus to maturity. *Animal* 12 s19–s26. 29551096 10.1017/S1751731118000460

[B32] Memczak S. Jens M. Elefsinioti A. Torti F. Krueger J. Rybak A. (2013). Circular RNAs are a large class of animal RNAs with regulatory potency. *Nature* 495 333–338. 23446348 10.1038/nature11928

[B33] Nolte W. Weikard R. Brunner R. M. Albrecht E. Hammon H. M. Reverter A. (2020). Identification and Annotation of Potential Function of Regulatory Antisense Long Non-Coding RNAs Related to Feed Efficiency in *Bos taurus* Bulls. *Int. J. Mol. Sci.* 21:3292. 10.3390/ijms21093292 PMC7247587 32384694

[B34] Nolte W. Weikard R. Brunner R. M. Albrecht E. Hammon H. M. Reverter A. (2019). Biological Network Approach for the Identification of Regulatory Long Non-Coding RNAs Associated With Metabolic Efficiency in Cattle. *Front. Genet.* 10:1130. 10.3389/fgene.2019.01130 31824560 PMC6883949

[B35] Pamudurti N. R. Bartok O. Jens M. Ashwal-Fluss R. Stottmeister C. Ruhe L. (2017). Translation of CircRNAs. *Mol. Cell* 66 9–21e27. 28344080 10.1016/j.molcel.2017.02.021 PMC5387669

[B36] Picut C. A. Ziejewski M. K. Stanislaus D. (2018). Comparative Aspects of Pre- and Postnatal Development of the Male Reproductive System. *Birth Defects Res.* 110 190–227. 29063715 10.1002/bdr2.1133

[B37] Pig (2021). *Pig.* Cambridge: Ensembl. http://www.ensembl.org/Sus_scrofa/Info/Index

[B38] Ragan C. Goodall G. J. Shirokikh N. E. Preiss T. (2019). Insights into the biogenesis and potential functions of exonic circular RNA. *Sci. Rep.* 9:2048. 10.1038/s41598-018-37037-0 PMC6376117 30765711

[B39] Rawlings N. Evans A. C. Chandolia R. K. Bagu E. T. (2008). Sexual maturation in the bull. *Reprod. Domest. Anim.* 43 (Suppl. 2), 295–301. 18638138 10.1111/j.1439-0531.2008.01177.x

[B40] Robic A. Kühn C. (2020). Beyond Back Splicing, a Still Poorly Explored World: Non-Canonical Circular RNAs. *Genes* 11:1111. 10.3390/genes11091111 PMC7565381 32972011

[B41] Robic A. Demars J. Kühn C. (2020). In-Depth Analysis Reveals Production of Circular RNAs from Non-Coding Sequences. *Cells* 9:1806. 10.3390/cells9081806 PMC7464727 32751504

[B42] Robic A. Faraut T. Djebali S. Weikard R. Feve K. Maman S. (2019). Analysis of pig transcriptomes suggests a global regulation mechanism enabling temporary bursts of circular RNAs. *RNA Biol.* 16 1190–1204. 31120323 10.1080/15476286.2019.1621621 PMC6693536

[B43] Robic A. Feve K. Riquet J. Prunier A. (2016). Transcript levels of genes implicated in steroidogenesis in the testes and fat tissue in relation to androstenone accumulation in fat of pubertal pigs. *Domest. Anim. Endocrinol.* 57 1–9. 27285831 10.1016/j.domaniend.2016.03.008

[B44] Robinson M. D. McCarthy D. J. Smyth G. K. (2010). edgeR: a Bioconductor package for differential expression analysis of digital gene expression data. *Bioinformatics* 26 139–140. 19910308 10.1093/bioinformatics/btp616 PMC2796818

[B45] Salzman J. Gawad C. Wang P. L. Lacayo N. Brown P. O. (2012). Circular RNAs are the predominant transcript isoform from hundreds of human genes in diverse cell types. *PLoS One* 7:e30733. 10.1371/journal.pone.0030733 22319583 PMC3270023

[B46] Sheep (2021). *Sheep.* Cambridge: Ensembl. http://www.ensembl.org/Ovis_aries_rambouillet/Info/Index

[B47] Soumillon M. Necsulea A. Weier M. Brawand D. Zhang X. Gu H. (2013). Cellular source and mechanisms of high transcriptome complexity in the mammalian testis. *Cell Rep.* 3 2179–2190. 23791531 10.1016/j.celrep.2013.05.031

[B48] Stoll L. Rodriguez-Trejo A. Guay C. Brozzi F. Bayazit M. B. Gattesco S. (2020). A circular RNA generated from an intron of the insulin gene controls insulin secretion. *Nat. Commun.* 11:5611. 10.1038/s41467-020-19381-w PMC7644714 33154349

[B49] Suenkel C. Cavalli D. Massalini S. Calegari F. Rajewsky N. (2020). A Highly Conserved Circular RNA Is Required to Keep Neural Cells in a Progenitor State in the Mammalian Brain. *Cell Rep.* 30 2170–2179e2175. 32075758 10.1016/j.celrep.2020.01.083

[B50] Taanman J. W. (1999). The mitochondrial genome: structure, transcription, translation and replication. *Biochim. Biophys. Acta* 1410 103–123. 10076021 10.1016/s0005-2728(98)00161-3

[B51] Taggart A. J. Lin C. L. Shrestha B. Heintzelman C. Kim S. Fairbrother W. G. (2017). Large-scale analysis of branchpoint usage across species and cell lines. *Genome Res.* 27 639–649. 28119336 10.1101/gr.202820.115 PMC5378181

[B52] Wang P. L. Bao Y. Yee M. C. Barrett S. P. Hogan G. J. Olsen M. N. (2014). Circular RNA is expressed across the eukaryotic tree of life. *PLoS One* 9:e90859. 10.1371/journal.pone.0090859 24609083 PMC3946582

[B53] Xiao M. S. Ai Y. Wilusz J. E. (2020). Biogenesis and Functions of Circular RNAs Come into Focus. *Trends Cell Biol.* 30 226–240. 31973951 10.1016/j.tcb.2019.12.004 PMC7069689

[B54] Yang W. Zhao F. Chen M. Li Y. Lan X. Yang R. (2020). Identification and characterization of male reproduction-related genes in pig (*Sus scrofa*) using transcriptome analysis. *BMC Genomics* 21 381. 10.1186/s12864-020-06790-w 32487021 PMC7268776

[B55] Zeng X. Lin W. Guo M. Zou Q. (2017). A comprehensive overview and evaluation of circular RNA detection tools. *PLoS Comput. Biol.* 13:e1005420. 10.1371/journal.pcbi.1006158 28594838 PMC5466358

[B56] Zhang J. Chen S. Yang J. Zhao F. (2020). Accurate quantification of circular RNAs identifies extensive circular isoform switching events. *Nat. Commun.* 11:90. 10.1038/s41467-019-13840-9 PMC6941955 31900416

[B57] Zhang X. O. Dong R. Zhang Y. Zhang J. L. Luo Z. Zhang J. (2016). Diverse alternative back-splicing and alternative splicing landscape of circular RNAs. *Genome Res.* 26 1277–1287. 27365365 10.1101/gr.202895.115 PMC5052039

[B58] Zhang Y. Zhang X. O. Chen T. Xiang J. F. Yin Q. F. Xing Y. H. (2013). Circular intronic long noncoding RNAs. *Mol. Cell* 51 792–806. 24035497 10.1016/j.molcel.2013.08.017

[B59] Zhao Q. Liu J. Deng H. Ma R. Liao J. Y. Liang H. (2020). Targeting Mitochondria-Located circRNA SCAR Alleviates NASH via Reducing mROS Output. *Cell* 183 76–93e22. 32931733 10.1016/j.cell.2020.08.009

[B60] Zhou T. Xie X. Li M. Shi J. Zhou J. Knox K. (2018). Rat BodyMap transcriptomes reveal unique circular RNA features across tissue types and developmental stages. *RNA* 24 1443–1456. 30093490 10.1261/rna.067132.118 PMC6191709

[B61] Zucko D. Boris-Lawrie K. (2020). Circular RNAs Are Regulators of Diverse Animal Transcriptomes: One Health Perspective. *Front. Genet.* 11:999. 10.3389/fgene.2020.00999 33193584 PMC7531264

