# Supplementary material for: Comparative Analysis of the Circular Transcriptome in Muscle, Liver, and Testis in Three Livestock Species
Source: Front Genet. 2021 May 10;12:665153. doi: 10.3389/fgene.2021.665153 (PMC8141914; doi:10.3389/fgene.2021.665153)
Supplement: Supplementary File 1 — Some features relative to circRNAs characterization (CE2 + CIRI2). [file Data_Sheet_1.ZIP › Add_Doc.pdf]

CIRI2+CE > 4 BSJs (CIRIquant evaluation)

	Cattle	bta_liver	bta_muscle	bta_testis
		bta_liver_1-15	bta_muscle_1-12	bta_testis_1-6
Reads	364.8 Gb	162.8 Gb	111.6 Gb	90.4 Gb
	3,47 10^9 Reads uniq. Map.	1,69 10^9 Reads uniq. Map.	1,27 10^9 Reads uniq. Map.	0,51 10^9 Reads uniq. Map.
**circRNAs (CE2+CIRI2)**	12,588 exonic and 6 ciRNAs from 4,631 genes	4,825 exonic circRNAs from 2,386 genes	1,813 exonic circRNAs from 1,183 genes	10,808 exonic circRNAs from 4,212 genes

	Pig	ssc_liver	ssc_muscle	ssc_testis
		ssc_liver_4-10	ssc_muscle_2-4	ssc_testis_2-10
Reads	332.3 Gb	72.65 Gb	87.8 Gb	171.8 Gb
	2,77 10^9 Reads uniq. Map.	0,534 10^9 Reads uniq. Map.	0,486 10^9 Reads uniq. Map.	1,75 10^9 Reads uniq. Map.
**circRNAs (CE2+CIRI2)**	14,137 exonic and 1 ciRNA 4,479 genes	3,260 exonic circRNAs from 1,798 genes	2,986 exonic circRNAs from 1,729 genes	12,335 exonic circRNAs from 4,034 genes

	Sheep	oar_liver	oar_muscle	oar_testis
		oar_liver_1-6	oar_muscle_1-6	oar_testis_1-3
Reads	465.7 Gb	181.6 Gb	191.2 Gb	92.9 Gb
	2,56 10^9 Reads uniq. Map.	1,03 10^9 Reads uniq. Map.	0,998 10^9 Reads uniq. Map.	0,533 10^9 Reads uniq. Map.
**circRNAs (CE2+CIRI2)**	5,555 exonic and 3 ciRNAs 2,793 genes	3,521 exonic circRNAs from 2,086 genes	1,889 exonic circRNAs from 1,252 genes	3,089 exonic circRNAs from 1,748 genes

Add_Doc-A

Some features relative to circRNAs characterization (CE2+CIRI2)

ciRNA: Although the term "ciRNA" is the one proposed for intronic circular RNA by Zhang et al (Ref-6), the location of the 3' junction of these circRNAs must be analyzed before to consider them as intronic circRNA.(both junctions fall within a single intron, the 5' junction corresponding to the intron donor site and the 3' junction located not further than 60 bp away from the intron acceptor site)

Datasets	Number of reads uniquely mapped on reference genome	Number of exonic circRNA characterized (CE2+CIRI2)	Number of exonic circRNA/million of reads uniq. Mapped
bta_liver_1	124 941 133	1870	14.97
bta_liver_2	108 265 643	1515	13.99
bta_liver_3	124 419 909	1735	13.95
bta_liver_4	117 590 496	2114	17.98
bta_liver_5	123 909 876	2056	16.59
bta_liver_6	133 773 221	2197	16.4
bta_liver_7	113 891 102	1638	14.38
bta_liver_8	113 210 410	1528	13.50
bta_liver_9	113 477 872	1834	16.16
bta_liver_10	131 717 410	2022	15.35
bta_liver_11	128 272 440	1797	14.01
bta_liver_12	126 463 887	2146	16.97
bta_liver_13	56 951 972	513	9.01
bta_liver_14	105 755 600	825	7.80
bta_liver_15	64 512 691	545	8.45
bta_muscle_1	113 381 027	677	5.97
bta_muscle_2	97 113 568	533	5.49
bta_muscle_3	115 058 742	771	6.70
bta_muscle_4	100 096 577	812	8.11
bta_muscle_5	96 359 457	487	5.05
bta_muscle_6	107 768 249	894	8.30
bta_muscle_7	98 737 788	391	3.96
bta_muscle_8	120 189 093	556	4.63
bta_muscle_9	93 954 158	427	4.55
bta_muscle_10	104 416 665	577	5.53
bta_muscle_11	111 199 262	773	6.95
bta_muscle_12	115 499 211	690	5.97
bta_testis_1	77 979 988	6070	77.84
bta_testis_2	76 466 706	5148	67.32
bta_testis_3	96 607 688	5631	58.29
bta_testis_4	85 542 458	2794	32.66
bta_testis_5	88 838 087	4108	46.24
bta_testis_6	87 177 880	3625	41.58

Datasets	Number of reads uniquely mapped on reference genome	Number of exonic circRNA characterized (CE2+CIRI2)	Number of exonic circRNA/million of reads uniq. Mapped
ssc_liver_5	118 770 167	1296	10.9
ssc_liver_6	111 512 647	1201	10.8
ssc_liver_7	129 999 205	1625	12.5
ssc_liver_8	61 242 560	1473	24.1
ssc_liver_9	67 841 139	1206	17.8
ssc_liver_10	44 608 460	788	17.7
ssc_muscle_2	126 184 501	1516	12.0
ssc_muscle_3	165 580 265	1841	11.1
ssc_muscle_4	193 811 628	1597	8.2
ssc_testis_2	147 453 222	7189	48.8
ssc_testis_3	147 678 932	5697	38.6
ssc_testis_4	134 827 093	4951	36.7
ssc_testis_5	109 679 267	3777	34.4
ssc_testis_6	132 017 185	5240	39.7
ssc_testis_7	133 144 301	5108	38.4
ssc_testis_8	389 212 510	2977	7.6
ssc_testis_9	314 763 377	3030	9.6
ssc_testis_10	245 682 717	2348	9.6

Datasets	Number of reads uniquely mapped on reference genome	Number of exonic circRNA characterized (CE2+CIRI2)	Number of exonic circRNA/million of reads uniq. Mapped
oar_liver_1	169 450 894	1228	7.2
oar_liver_2	140 676 363	1321	9.4
oar_liver_3	188 682 040	1719	9.1
oar_liver_4	202 992 205	1311	6.5
oar_liver_5	183 245 363	1530	8.3
oar_liver_6	144 362 786	846	5.9
oar_muscle_1	148 371 363	754	5.1
oar_muscle_2	154 484 113	745	4.8
oar_muscle_3	191 908 806	852	4.4
oar_muscle_4	190 304 564	971	5.1
oar_muscle_5	128 784 967	550	4.3
oar_muscle_6	184 107 737	697	3.8
oar_testis_1	174 702 244	1317	7.5
oar_testis_2	119 000 789	1108	9.3
oar_testis_3	239 468 827	2074	8.7

Add_Doc-B

Characterization of exonic circRNAs performed by CE2+CIRI2

					Statistical analysis								
					pairwise comparison				Conclusion				
		**dataset-1**	**dataset-2**		**mean-1** (1)	**mean-2** (1)		**p-value**					
liver	muscle	bta_liver_1-12	bta_muscle_1-12	N. of circRNAs annotated as exonic circRNAs CD	10.62	3.77	yes	3.1 E-09	**Cattle**	**liver**	**>**	**muscle**	**exonic circRNAs**
liver	muscle	bta_liver_1-12	bta_muscle_1-12	N. of CCRs associated to exonic circRNAs CD	156.09	52.46	yes	1.8 E-08					
liver	muscle	bta_liver_1-12	bta_muscle_1-12	N. of circRNAs detected by CE2+CIRI2	15.36	5.93	yes	3.10 E-10					
2 days	13 months	bta_testis_1-3	bta_testis_4-6	N. of circRNAs annotated as exonic circRNAs CD	44.79	24.76	no	0.016	**cattle_testis**	**2 days**	**>**	**13 months**	**exonic circRNA**
2 days	13 months	bta_testis_1-3	bta_testis_4-6	N. of CCRs associated to exonic circRNAs CD	834.94	358.46	no	0.011					
2 days	13 months	bta_testis_1-3	bta_testis_4-6	N. of circRNAs detected by CE2+CIRI2	67.82	40.16	no	0.016					
liver	muscle	oar_liver_1-6	oar_muscle_1-6	N. of circRNA annotated as exonic circRNAs CD	5.00	2.94	yes	0.0067	**Sheep**	**liver**	**>**	**muscle**	**exonics circRNAs**
liver	muscle	oar_liver_1-6	oar_muscle_1-6	N. of CCRs associated to exonic circRNAs CD	63.50	36.51	yes	0.019					
liver	muscle	oar_liver_1-6	oar_muscle_1-6	N. of circRNAs detected by CE2+CIRI2	7.74	4.58	yes	0.0031					
liver	muscle	ssc_liver_5-7	ssc_muscle_2-4	N. of circRNAs annotated as exonic circRNAs CD	6.19	5.77	yes	0.45					
liver	muscle	ssc_liver_5-7	ssc_muscle_2-4	N. of CCRs associated to exonic circRNAs CD	96.11	76.92	yes	0.11	**Pig**	**no significative difference**			
liver	muscle	ssc_liver_5-7	ssc_muscle_2-4	N. of circRNAs detected by CE2+CIRI2	11.39	10.46	yes	0.47					

Add_Doc-C **All statistics relative to comparisons of the number of circRNAs**

(1) per million of reads uniquely mapped

All statistics relative to comparisons of the number of circRNAs

All p-values were provided for two-sided tests. Pairwise comparison (paired sample t-test): Results with $p<0.05$ were considered statistically significant.

Long non-coding genes	Long non-coding genes	Long non-coding genes	Long non-coding genes	Pseudogenes	Small non-coding genes
**Cattle**	**Sheep**	**Sheep**	**Sheep**		**Cattle**
ENSBTAG00000049855	ENSOARG00020020442	ENSOARG00020012448	ENSOARG00020000184		ENSBTAG00000044853
ENSBTAG00000051239	ENSOARG00020020695	ENSOARG00020012669	ENSOARG00020000255		
ENSBTAG00000052059	ENSOARG00020020699	ENSOARG00020012798	ENSOARG00020000806		
ENSBTAG00000052752	ENSOARG00020020744	ENSOARG00020012937	ENSOARG00020000895		
ENSBTAG00000053856	ENSOARG00020020791	ENSOARG00020013102	ENSOARG00020000904		
ENSBTAG00000054037	ENSOARG00020021149	ENSOARG00020013570	ENSOARG00020002007		
	ENSOARG00020021494	ENSOARG00020013980	ENSOARG00020002499		
	ENSOARG00020021582	ENSOARG00020014026	ENSOARG00020002514		
**Pig**	ENSOARG00020021588	ENSOARG00020014136	ENSOARG00020003164		
ENSSSCG00000003846	ENSOARG00020021630	ENSOARG00020014325	ENSOARG00020003863		
ENSSSCG00000007311	ENSOARG00020021825	ENSOARG00020014451	ENSOARG00020003933	**Pig**	**Pig**
ENSSSCG00000016379	ENSOARG00020021852	ENSOARG00020014763	ENSOARG00020004322	ENSSSCG00000007066	ENSSSCG00000025715
ENSSSCG00000021146	ENSOARG00020022108	ENSOARG00020014824	ENSOARG00020004437	ENSSSCG00000007191	
ENSSSCG00000035981	ENSOARG00020022250	ENSOARG00020015148	ENSOARG00020004935	ENSSSCG00000008996	
ENSSSCG00000036117	ENSOARG00020022464	ENSOARG00020015455	ENSOARG00020005056	ENSSSCG00000014877	
ENSSSCG00000036428	ENSOARG00020022957	ENSOARG00020015556	ENSOARG00020005400	ENSSSCG00000022947	
ENSSSCG00000038228	ENSOARG00020023339	ENSOARG00020015727	ENSOARG00020005533	ENSSSCG00000031738	
ENSSSCG00000039815	ENSOARG00020023457	ENSOARG00020016048	ENSOARG00020005672	ENSSSCG00000040835	
ENSSSCG00000041097	ENSOARG00020023804	ENSOARG00020016135	ENSOARG00020005773		
ENSSSCG00000041317	ENSOARG00020024174	ENSOARG00020016223	ENSOARG00020005865		
ENSSSCG00000041715	ENSOARG00020024528	ENSOARG00020016287	ENSOARG00020006025	**Sheep**	
ENSSSCG00000042491	ENSOARG00020024783	ENSOARG00020016518	ENSOARG00020006139	ENSOARG00020024838	
ENSSSCG00000042814	ENSOARG00020025201	ENSOARG00020017120	ENSOARG00020006451	ENSOARG00020025782	
ENSSSCG00000043931	ENSOARG00020025437	ENSOARG00020017255	ENSOARG00020006749		
ENSSSCG00000045125	ENSOARG00020025496	ENSOARG00020017265	ENSOARG00020007083		
ENSSSCG00000045227	ENSOARG00020025648	ENSOARG00020018001	ENSOARG00020007804		
ENSSSCG00000045693	ENSOARG00020025737	ENSOARG00020018837	ENSOARG00020007978		
ENSSSCG00000045708	ENSOARG00020025836	ENSOARG00020018958	ENSOARG00020008047		
ENSSSCG00000045780	ENSOARG00020025867	ENSOARG00020019642	ENSOARG00020008238		
ENSSSCG00000046272	ENSOARG00020025953	ENSOARG00020019736	ENSOARG00020008835		
ENSSSCG00000048308		ENSOARG00020019824	ENSOARG00020008956		
ENSSSCG00000048397		ENSOARG00020020155	ENSOARG00020009200		
ENSSSCG00000048531			ENSOARG00020009892		
ENSSSCG00000049333			ENSOARG00020010148		
ENSSSCG00000050004			ENSOARG00020010183		
ENSSSCG00000050424			ENSOARG00020010528		
ENSSSCG00000050772			ENSOARG00020010866		
ENSSSCG00000050893			ENSOARG00020011247		
ENSSSCG00000050927			ENSOARG00020011726		
ENSSSCG00000051601			ENSOARG00020012124		
ENSSSCG00000051610			ENSOARG00020012139		

Add_Doc-D

List of non-coding genes considered as able to produce exonic circRNAs (CE2+CIRI2 and CD)

Cattle		bta_liver		bta_muscle		bta_testis
		bta_liver_1-15		bta_muscle_1-12		bta_testis_1-6
**Top-3 of genes capable of producing the most distinct circRNAs**	23	ENSBTAG00000003746	30	ENSBTAG00000006907	30	ENSBTAG00000008449
	18	ENSBTAG00000018138	12	ENSBTAG00000011392	25	ENSBTAG00000012357
	17	ENSBTAG00000023177	10	ENSBTAG00000014581	25	ENSBTAG00000009665
		ENSBTAG00000003746 (X23) SCP2		ENSBTAG00000006907 (X30) NEB		ENSBTAG00000008449 (X30) DNAH14

Pig		ssc_liver		ssc_muscle		ssc_testis
		ssc_liver_4-10		ssc_muscle_2-4		ssc_testis_2-10
**Top-3 of genes capable of producing the most distinct circRNAs**	19	ENSSSCG00000029837	16	ENSSSCG00000016397	41	ENSSSCG00000011340
	16	ENSSSCG00000035347	15	ENSSSCG00000006137	31	ENSSSCG00000004979
	15	ENSSSCG00000002266	13	ENSSSCG00000004979	30	ENSSSCG00000038125
					30	ENSSSCG00000015807
		ENSSSCG00000029837 (X19) VWA8		ENSSSCG00000016397 (X16) NEB		ENSSSCG00000011340 (X41) SMARCC1

Sheep		oar_liver		oar_muscle		oar_testis
		oar_liver_1-6		oar_muscle_1-6		oar_testis_1-3
**Top-3 of genes capable of producing the most distinct circRNAs**	14	ENSOARG00020009563	19	ENSOARG00020024247	18	ENSOARG00020019907
	13	ENSOARG00020005673	10	ENSOARG00020006228	14	ENSOARG00020016617
	11	ENSOARG00020015171	9	ENSOARG00020013638	12	ENSOARG00020023485
	11	ENSOARG00020013638			12	ENSOARG00020003056
		ENSOARG00020009563 (X13) CEP112		ENSOARG00020024247 (X19) NEB		ENSOARG00020019907 (X18) RYR2

Add_Doc-E **Genes able to produce multiple exonic circRNAs (CE2+CIRI2)**
